# Impacts of aging and fluid shear stress on vascular endothelial metabolism and atherosclerosis development

**DOI:** 10.1186/s12929-025-01177-z

**Published:** 2025-09-01

**Authors:** Wei-Li Wang, Yu-Tsung Shih, Shu-Yi Wei, Jeng-Jiann Chiu

**Affiliations:** 1 Institute of Cellular and System Medicine, National Health Research Institutes, Miaoli, Taiwan; 2 Department of Anatomy and Cell Biology, School of Medicine, College of Medicine, Taipei Medical University, Taipei, Taiwan; 3 College of Medical Science and Technology, Taipei Heart Institute, Taipei Medical University, Taipei, Taiwan; 4 Institute of Biomedical Engineering, National Tsing Hua University, Hsinchu, Taiwan

**Keywords:** Endothelial cell metabolism, Aging, Shear stress, Atherosclerosis, Metabolic reprogramming

## Abstract

Aging is the foremost risk factor for metabolic syndrome and atherosclerosis, which is a principal cause of cardiovascular diseases (CVDs). Vascular endothelial cells (ECs), which line the vascular intima, play a central role in maintaining vascular homeostasis. Their dysfunction, marked by impaired barrier function, inflammation, and metabolic dysregulation, constitutes an early and pivotal event in atherogenesis. As key sensors of hemodynamic forces, ECs are constantly exposed to blood flow-induced shear stress, which exert divergent effects on metabolism depending on the flow pattern. Laminar flow with relatively high shear stress (LS), as a critical atheroprotective factor, maintains EC quiescence and promotes anti-inflammatory responses and antioxidant defense, whereas disturbed flow with low and oscillatory shear stress (OS), induces the athero-susceptible signaling network to activate glycolysis and inflammation in ECs. While genetic, epigenetic, and molecular signaling mechanisms in EC physiology and pathophysiology have been extensively explored, the crucial role of EC metabolism in EC dysfunction and atherogenesis remains largely understudied. By serving as precursors, intermediates, and end products of cellular processes, metabolites offer a dynamic snapshot of endothelial metabolic states under both physiological and pathophysiological conditions. With aging, ECs undergo profound metabolic reprogramming, including disrupted glycolysis, mitochondrial dysfunction, and altered redox homeostasis. In healthy vasculature, ECs maintain quiescence and metabolic homeostasis, primarily relying on glycolysis for energy. With aging, the gradual accumulation of atherosclerotic risk factors, including oxidative stress, inflammation, dyslipidemia, and hyperglycemia, drives metabolic reprogramming in ECs, particularly in regions exposed to disturbed flow with OS, ultimately leading to EC dysfunction and atherosclerosis. This review summarizes recent advances in age-related metabolic reprogramming in ECs and its contribution to atherosclerosis, particularly focusing on the dysregulation of glycolysis, fatty acid metabolism, amino acid metabolism, and mitochondrial respiration induced by age and fluid shear stress. This review also outlines recent methodologies for profiling EC metabolism, and discusses potential therapeutic applications of targeting EC metabolism to prevent or delay the development of atherosclerosis.

## Background

Aging is the primary risk factor for age-related diseases, particularly cardiovascular diseases (CVDs) such as coronary artery disease (CAD), stroke, peripheral artery disease, heart failure, and aneurysms. With advancing age, factors like oxygen radicals, inflammation, elevated lipid levels, mechanical forces, and glucose progressively accumulate, accelerating vascular aging and promoting atherosclerosis [1]. Notably, approximately 40% of deaths in individuals over 65 years are attributed to atherosclerosis and its complications [2]. Atherosclerosis involves plaque buildup within arterial walls, leading to vascular narrowing, stiffness, and ischemic complications [1, 3].

The earliest detectable change in atherosclerotic lesion development occurs at the endothelial layer. Endothelial cells (ECs), lining the intimal surface, maintain vascular tone, barrier function, immune quiescence, and metabolic balance. These cells are constantly exposed to circulating biochemical signals and mechanical stimuli, including blood flow-induced shear stress, stretching, and pressure [4, 5]. Among these risk factors, blood fluid shear stress is an important factor regulating EC physiology and pathophysiology and atherosclerosis progression. Laminar flow and its associated shear stress (LS), which is characterized by steady, unidirectional flow, promotes healthy EC function and is atheroprotective. In contrast, disturbed flow with low and oscillatory shear stress (OS), that occurs at arterial branches and curvatures, can lead to EC dysfunction and promote atherosclerosis. Importantly, atherosclerotic plaque preferentially develops in areas of disturbed flow [6]. The EC layer is crucial for maintaining vascular homeostasis by regulating the balance between vasodilators and vasoconstrictors, anti- and proinflammatory factors, as well as antioxidants and oxidants [7]. However, with aging, these EC homeostatic functions progressively diminish [8]. Senescent ECs accumulate in arterial tissues throughout the human lifespan and are positively associated with atherosclerosis [9, 10]. Aging is also correlated with arterial dilation and stiffening, which reduce the mean shear stress, particularly decreasing antegrade (forward) shear stress [11]. Increased irregularities in blood flow patterns result in elevated levels of disturbed shear stress [12]. These age-related changes alter the hemodynamics of blood flow, leading to less-favorable shear stress distributions and causing EC dysfunction and increased susceptibility to atherosclerosis. [6] While extensive studies have explored the genetic, epigenetic, and post-transcriptional pathways contributing to EC dysfunction [5, 6, 13–16], the contribution of cellular metabolism to vascular aging and atherosclerosis is comparatively understudied. Unlike genes, the number of known metabolites is relatively low (approximately 6500 distinct small molecules compared to around 25,000 genes, 100,000 transcripts, and 1 million proteins), allowing for high-resolution network analyses of cellular processes.

**Fig. 1:**
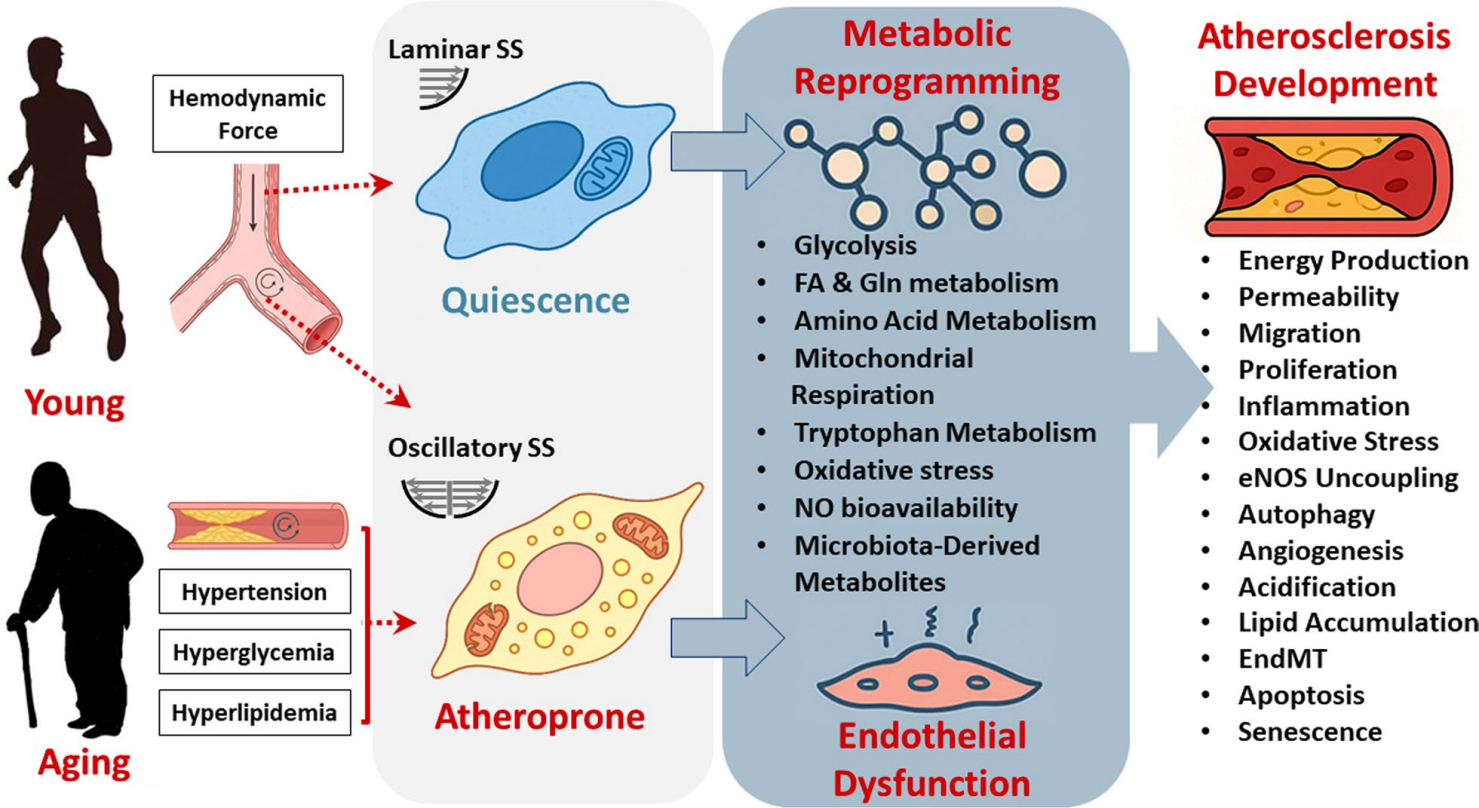
Integrated model of aging, shear stress, and metabolic reprogramming in EC dysfunction and atherosclerosis. This schematic summarizes the interplay between aging-related factors (e.g., hypertension, hyperglycemia, and hyperlipidemia) and hemodynamic forces (laminar vs. oscillatory shear stress) in modulating EC metabolism. Aging and disturbed flow synergistically drive EC metabolic reprogramming—enhancing glycolysis, reactive oxygen species (ROS), and impairing nitric oxide (NO) bioavailability—thereby promoting EC dysfunction and atherosclerotic plaque development

Despite their proliferative arrest, senescent cells exhibit a metabolically active state characterized by an altered glycolytic flux, perturbed lipid homeostasis, mitochondrial dysfunction, and a disrupted nicotinamide adenine dinucleotide (NAD +) redox balance, all of which contribute to vascular damage. In the context of atherosclerosis, a comprehensive systems-level analysis of EC metabolic reprogramming provides an important avenue for developing targeted therapeutic interventions aimed at mitigating age-related vascular pathologies [3]. Previous studies documented aging’s impacts on metabolism and EC’s role in atherosclerosis [1, 3, 17]. This review delves into the latest research, focusing on how age- and fluid shear stress-related metabolic changes within ECs contribute to the formation and progression of atherosclerosis (Fig. 1). In addition, current and potential interventions to modulate EC metabolism for preventing or delaying atherosclerosis onset are discussed.

## Metabolism in quiescent, activated, and dysfunctional ECs

Cellular metabolism serves three primary functions: energy production, biomass synthesis, and redox homeostasis. It also supports additional processes, such as waste elimination and other metabolic activities [18]. Although cellular metabolism has been studied for over a century, EC metabolism has only recently garnered significant attention. In healthy blood vessels, ECs exhibit low metabolic activity and remain in a quiescent state that is stable and maintains the vascular integrity, regulates permeability, and prevents unnecessary clotting and inflammation [19, 20]. With advancing age and the gradual accumulation of stimuli such as inflammatory cytokines, growth factors, or physical stress, ECs shift into an activated state characterized by heightened metabolic activity [3, 21]. Under chronic stress, dysfunctional ECs are pathologically altered into cells associated with increased permeability, chronic inflammation, senescence, and metabolic reprogramming that promote atherogenesis. Furthermore, metabolic disturbances were shown to influence key pathological features of atherosclerosis, including inflammation, increased permeability, and morphological changes such as the endothelial-to-mesenchymal transition (EndMT) [17]. Investigations into metabolic features of aging ECs have provided valuable insights into the pathogenesis of CVDs. This section provides a concise overview of glycolysis, fatty acids (FAs), amino acid metabolism, and mitochondrial respiration in ECs under normal and atherogenic conditions. Although numerous studies have been conducted on EC metabolism, most were performed on two-dimensional (2D)-cultured cells, which fail to replicate the 3D physiological environment. Moreover, the metabolites present in blood differ from those in culture media used for in vitro studies. As a result, the findings might not fully reflect the conditions of vascular ECs in different organs and tissues in vivo. Future in vivo studies are necessary to enhance or refine the current research findings.

### Glycolysis

Glycolysis, the metabolic breakdown of glucose, serves as the central and an indispensable energy source in ECs, providing ATP and biosynthetic precursors necessary for cellular maintenance, proliferation, and responses to stress. Glucose enters ECs via glucose transporters (GLUTs), where it is metabolized into pyruvate through a series of enzymatic reactions, generating two ATP and two nicotinamide adenine dinucleotide hydrogen (NADH) molecules. The process is tightly regulated by rate-limiting enzymes including hexokinase 2 (HK2), that phosphorylates glucose to glucose-6-phosphate; phosphofructokinase (PFK), which converts fructose-6-phosphate into fructose 1,6-bisphosphate and PFK/fructose bisphosphatase 3 (PFKFB3), an allosteric activator of PFK that becomes particularly important in glycolysis conditions and pathological angiogenesis [22–24].

**Fig. 2:**
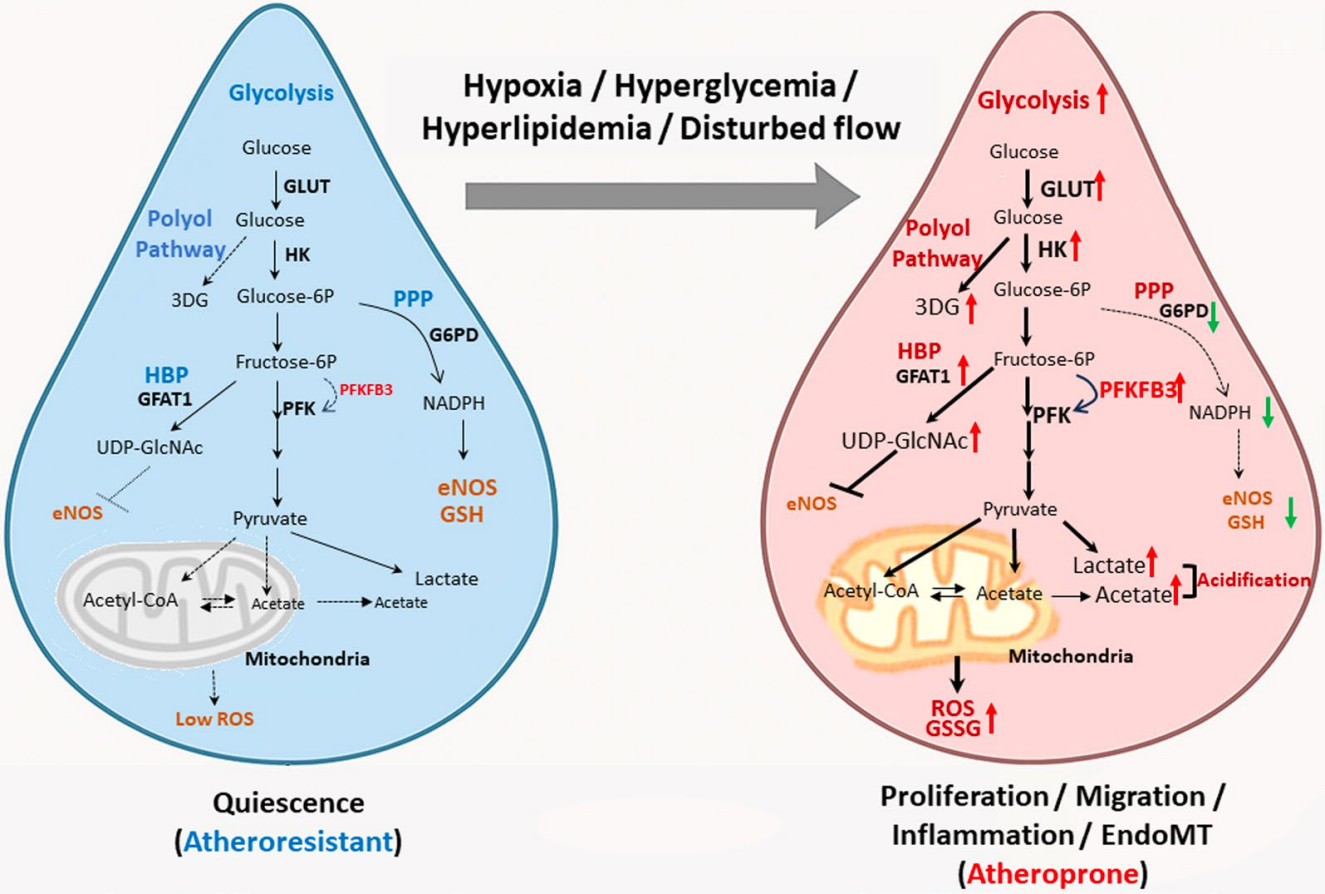
Glycolytic remodeling and redox imbalance in quiescent versus aged or inflamed ECs. Quiescent ECs under laminar shear stress exhibit controlled glycolysis, low reactive oxygen species (ROS) levels, and balanced nicotinamide adenine dinucleotide phosphate (NADPH) production via the pentose phosphate pathway (PPP). In contrast, aged or inflamed ECs shift toward enhanced glycolysis and hexosamine biosynthesis pathway (HBP) activity, leading to ROS accumulation, glutathione (GSH) depletion, and impaired nitric oxide (NO) signaling. Pathways shown with a dashed line in the figure have minimal activity in healthy ECs. *GLUT* glucose transporter, *HK* hexokinase, *3DG* 3-deoxyglucosone, *PPP* pentose phosphate pathway, *G6PD* glucose-6-phosphate dehydrogenase, *eNOS* endothelial nitric oxide synthase, *GSH* glutathione, *GSSG* glutathione disulfide (oxidative GSH), *HBP* hexosamine biosynthesis pathway, *GFAT1* glutamine fructose-6-phosphate amidotransferase, *UDP-GlciNAc* uridine diphosphate N-acetylglucosamine, *PFK* phosphofructokinase, *PFKFB3* 6-phosphofructo-2-kinase/fructose-2,6-bisphosphatase 3, *ROS* reactive oxygen species

In the presence of oxygen, glycolysis-generating pyruvate enters mitochondria for oxidative phosphorylation (OXPHOS); under anaerobic conditions, it is converted to lactate by lactate dehydrogenase (LDH). Interestingly, despite the presence of oxygen, quiescent ECs predominantly rely on glycolysis to generate approximately 85% of their ATP and bypass OXPHOS, likely due to their low mitochondrial content [25–29]. This metabolic profile offers several advantages. First, glycolysis is oxygen-independent, thus providing an advantage in hypoxic environments, such as poorly perfused tissues or during inflammation [25, 30]. Moreover, when glucose is abundant, glycolysis generates ATP more rapidly than OXPHOS [31]. Second, minimal reliance on OXPHOS reduces reactive oxygen species (ROS) production, thereby protecting ECs from oxidative damage. Additionally, glycolytic side pathways, such as the pentose phosphate pathway (PPP), produce NADH phosphate (NADPH) for regenerating glutathione (GSH), a vital antioxidant [27, 28]. Finally, by minimizing oxygen consumption, ECs preserve oxygen availability for underlying tissues [19]. However, during aging and atherosclerotic conditions, EC glucose metabolism becomes dysregulated, contributing to EC dysfunction (Fig. 2).

#### Dysregulation of glycolysis in aging ECs and atherosclerosis

Senescent ECs exhibit upregulation of glycolysis [19, 32], which may initially support energy requirements of migration and proliferation [19], but becomes maladaptive under pathological conditions. Hyperactivation of glycolysis contributes to dysfunctional EC proliferation, aberrant migration, and pathological angiogenesis, all of which promote plaque formation and instability [33]. 6-Phosphofructo-2-kinase/fructose-2,6-bisphosphatase 3 (PFKFB3), a crucial glycolytic regulator, converts fructose-6-phosphate to fructose-2,6-bisphosphate (F2,6BP), enhancing PFK-1 activity and thus promoting EC proliferation, migration, and angiogenesis [23]. Moreover, elevated PFKFB3 expression is commonly observed in vulnerable plaque regions [34]. Studies showed that inhibition of PFKFB3 partially reduces glycolysis, and its inhibition was shown to normalize EC behavior, mitigate pathological angiogenesis, and promote atherosclerotic plaque stability [24, 34–36]. Moreover, PFKFB3-mediated glycolysis promotes EC inflammation, particularly in response to atherogenic stimuli such as lipoprotein(a) (Lp(a)) and tumor necrosis factor (TNF)-α [37, 38], and contributes to EndMT, a key event in atherogenesis [39]. Hypo-glycolysis metabolism in ECs may also contribute to endothelial dysfunction in atherosclerosis. Xu et al. found that microRNA (miR)-143, which inhibits key glycolytic enzymes like HK2, LDHA, and PKM2, was upregulated in atherosclerotic plaque. Overexpression of miR-143 significantly decreases glucose consumption and ATP formation. This energy deficit directly impairs crucial EC functions. [40]. Similarly, Yang et al. demonstrated that disturbed flow activates the protein kinase, AMP-activated, alpha 1 (PRKAA1)/AMP-activated protein kinase α1 (AMPKα1)-hypoxia-inducible factor α (HIFα)-glycolysis axis that supports EC proliferation, and inhibition of PRKAA1 accelerates atherosclerosis in ApoE^−/−^ mice [41]. These results suggest that both hyper- and hypo-glycolysis disrupt EC homeostasis, and a glycolytic balance is essential for vascular health and atherogenesis. However, the detailed mechanisms by which glycolysis and glycolytic intermediates mediate atherosclerosis progression remain incompletely understood.

#### Glycolysis-derived intermediates in EC dysfunction and atherosclerosis

Lactate, derived from glycolysis and glutamine catabolism, functions as a crucial carbon source for cellular metabolism and a key signaling molecule [42]. Elevated lactate levels are associated with various inflammatory diseases and serve as prognostic indicators in CVDs [43–45]. In atherogenesis, enhanced glycolysis further increases lactate production and release [46], which leads to extracellular acidification, increases EC permeability, and accelerates atherosclerosis progression [47]. In addition to these indirect effects, lactate also directly modulates vascular inflammation and angiogenesis by activating nuclear factor (NF)-κB and HIF-1α pathways [48–52]. Xu et al. demonstrated that lactate promotes PFKFB3-driven angiogenesis through AKT phosphorylation [22], while lactate also induces EC permeability via extracellular signal-regulated kinase (ERK)-dependent activation of calpain1/2 to disrupt vascular endothelial (VE)-cadherin [53]. Moreover, lactate contributes to epigenetic reprogramming via histone lactylation. Zhang et al. demonstrated that glycolysis-derived lactate can modify histone by adding lactyl groups to become histone lysine (K) residues, a process known as "lactylation," which influences gene transcription and epigenetic modifications [54]. Dong et al. reported that lactate-dependent H3K18 lactylation (H3K18la) promotes the EndMT and atherosclerosis progression [55]. In addition to histones, lactate also induces lactylation of snail, a transforming growth factor (TGF)-β transcription factor that activates the TGF-β/Smad2 signaling pathway, further promoting the EndMT [56].

Interestingly, lactate may also have protective roles. Exercise is recognized as an atheroprotective factor and is known to generate significant amounts of lactate. Exercise-induced lactate activates G protein-coupled receptor 81 (GPR81), also known as hydroxy carboxylic acid receptor 1 and a selective lactate-sensing receptor, which is downregulated under atherogenic OS. Activation of GPR81 prevents OS-induced inflammation through the ERK5/Krüppel-like factor 2 (KLF2) signaling pathway [57]. In addition, lactate-induced lactylation of methylated CpG-binding protein 2 represses epiregulin/mitogen-activated protein kinase (MAPK) signaling, inhibiting EC inflammation and atherosclerosis progression [58]. Thus, lactate plays a dual role in atherosclerosis, being both a detrimental and potentially beneficial factor.

Glycolysis produces pyruvate, which typically enters mitochondria and is metabolized by pyruvate dehydrogenase (PDH) to acetyl-CoA, subsequently entering the tricarboxylic acid (TCA) cycle in physiological conditions. However, during hyperactive glucose metabolism, pyruvate can be atypically metabolized into free acetate instead of acetyl-CoA [59]. Zhu et al. demonstrated that this acetate accumulation promotes the EndMT, contributing to atherosclerosis [60]. Paradoxically, some research suggested that increasing acetyl-CoA levels via acetate supplementation might suppress TGF-β-induced EndoMT. This indicates a complex and context-dependent role for acetate [61]. Collectively, acetate may also exert beneficial effects in the aging endothelium by rescuing epigenetic homeostasis. These results indicate that acetate counteracts aging-associated endothelial dysfunction by epigenetic rescue.

Taken together, these findings highlight that dysregulated glycolytic intermediates, particularly acetate and lactate, play dual and context-dependent roles in modulating endothelial aging and atherosclerosis.

#### Side pathways of glycolysis: the pentose phosphate pathway (PPP), hexosamine biosynthesis pathway (HBP), and polyol pathway

In ECs, a portion of glucose enters alternative metabolic routes beyond classical glycolysis. These side pathways—namely the PPP, HBP, and polyol pathway—play critical roles in regulating redox balance, protein glycosylation, and metabolic stress responses.

- **Pentose phosphate pathway.** The PPP is the primary cellular source of NADPH, which supports reductive biosynthesis, antioxidant defense, and endothelial nitric oxide synthase (eNOS)-mediated nitric oxide (NO) production [62]. NADPH is also essential for glutathione reductase to regenerate reduced GSH, maintaining intracellular redox homeostasis [19, 63]. Glucose-6-phosphate dehydrogenase (G6PD), the enzyme of the PPP, was shown to enhance NADPH and NO production while reducing ROS in ECs under oxidative stress [64]. Conversely, G6PD inhibition lowers NADPH and NO levels, leading to EC dysfunction [39, 65, 66]. A G6PD deficiency is associated with increased cardiovascular risks [67, 68], particularly in elderly populations [69]. Nevertheless, the precise mechanisms through which the PPP and G6PD regulate EC dysfunction and atherogenesis during aging remain unclear and warrant further investigation.
- **Hexosamine biosynthesis pathway.** Within the HBP, fructose-6-phosphate is converted into glucosamine-6-phosphate and is subsequently metabolized into uridine diphosphate N-acetylglucosamine (UDP-GlcNAc), a crucial metabolite for *O-* and *N-*linked protein glycosylation, supporting various EC functions and cardiovascular pathology [70, 71]. HBP activity exerts context-dependent effects: acute activation may offer cardioprotection, whereas chronic HBP upregulation under hyperglycemia induces endoplasmic reticular (ER) stress, lipid accumulation, and inflammation [72]. Overexpression of glutamine:fructose-6-phosphate amidotransferase (GFAT), the rate-limiting enzyme of the HBP, was implicated in metabolic stress and atherosclerosis under a hyperglycemic condition [73]. Enhancing the HBP leads to increased UDP-GlcNAc and O-linked glycosylation, which impair eNOS activity [74]. Although emerging evidence suggests that both aging and chronic hyperglycemia activate the HBP in ECs, the direct impact of age-associated HBP remodeling on vascular dysfunction remains insufficiently characterized.
- **Polyol pathway.** Under physiological conditions, only approximately 3% of glucose enters the polyol pathway. However, in aging and hyperglycemic states, its activity is markedly enhanced. This pathway, catalyzed by aldose reductase (ALR2) and sorbitol dehydrogenase (SORD), converts glucose to fructose, consuming NADPH–a crucial antioxidant. Excess fructose may generate 3-deoxyglucosone (3-DG), a precursor for harmful advanced glycation end products (AGEs) [19, 75, 76]. AGEs not only suppress eNOS expression but also trigger oxidative stress via the MAPK/ERK pathways [77, 78] While largely inactive under normal conditions, hyperglycemia activates the polyol pathway in ECs, leading to detrimental consequences [79].

In summary, both aging and atherosclerosis promote increased glycolysis and the accumulation of intermediates and metabolites from glycolysis and its side pathways, leading to EC dysfunction. Elucidating the age-specific regulation of these pathways and their metabolite-mediated signaling mechanisms may provide novel targets for preventing or reversing endothelial dysfunction in atherosclerosis.

### Fatty acid (FA) metabolism

**Fig. 3:**
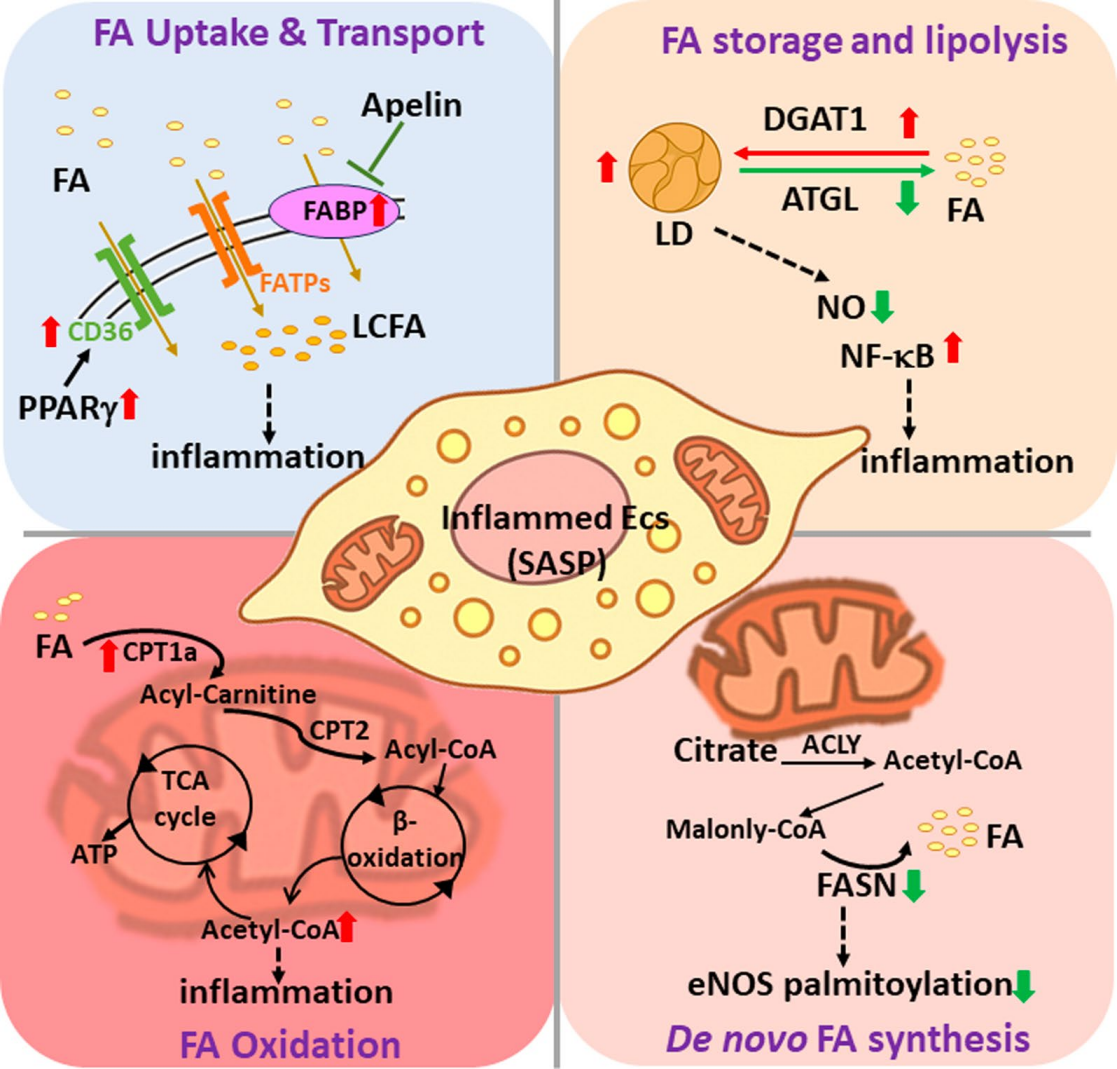
Dysregulation of FA metabolism in inflamed ECs. Aging and disturbed flow alter FA uptake, storage, oxidation, and de novo synthesis by ECs. Dysregulation of lipid transporters (e.g., cluster of differentiation 36 (CD36), FA transporter protein (FATP), and FA-binding protein (FABP)), mitochondrial β-oxidation (e.g., carnitine palmitoyl transferase 1a (CPT1a) and CPT2), lipolytic enzymes (e.g., adipose triglyceride lipase (ATGL) and diacylglycerol O-acyltransferase 1(DGAT1)), and FA synthetic enzymes (e.g., FA synthase (FASN)) contributes to lipid accumulation, inflammation, and the senescence-associated secretory phenotype (SASP) in inflamed vascular endothelium. *LCFA* long-chain fatty acid, *LD* lipid droplet

FA metabolism encompasses several processes, including FA uptake, transport, storage, oxidation, and de novo FA synthesis. FA metabolism plays a crucial role in modulating EC functions by regulating energy production, intracellular signaling, membrane synthesis, and inflammatory responses. Emerging evidence has highlighted a strong link between FA metabolism and cellular senescence [80]. Senescent cells often exhibit dysregulated lipid metabolism, characterized by the accumulation of free FAs (FFAs) and lipid droplets (LDs). Excessive FA storage contributes to oxidative stress and chronic inflammation, thereby accelerating senescence. Impaired mitochondrial FA oxidation (FAO) in senescent cells reduces ATP production, elevates ROS levels, and further promotes senescence. Enhanced de novo lipogenesis was observed in senescent cells, contributing to metabolic reprogramming and the proinflammatory senescence-associated secretory phenotype (SASP) (Fig. 3). Such FA metabolic disturbances contribute to the pathogenesis of multiple age-related diseases, including atherosclerosis, diabetes, and neurodegenerative disorders. Although the precise role of dysregulated FA metabolism in senescent ECs during atherosclerosis remains unclear, elucidating the underlying mechanism may offer new therapeutic strategies to mitigate vascular aging and atherosclerosis.

#### FA uptake and transport

In ECs, long-chain FAs (LCFAs, consisting of 12–20 carbons) are taken up from the circulation and transported across plasma membranes by a set of specific proteins, including lipoprotein lipase (LPL), glycosylphosphatidylinositol-anchored high-density lipoprotein-binding protein 1 (GPIHBP1), cluster of differentiation 36 (CD36), FA transport proteins (FATPs), and FA-binding proteins (FABPs) [81]. Among these, CD36 is the most well-characterized FA transporter. EC-specific CD36-knockout enhances glucose utilization, whereas a CD36/low-density lipoprotein receptor (LDLR) deficiency reduced atherosclerotic lesion formation in a murine model [82]. Similarly, FABPs, particularly FABP4, play essential roles in mediating endothelial-specific LCFA trafficking to specific intracellular sites for oxidation or storage in lipid homeostasis [83]. Key signaling pathways, including peroxisome proliferator-activated receptor γ (PPARγ) and Notch, regulate FABP4 expression to facilitate transendothelial FA transport [84, 85]. Conversely, downregulation of FABP4 by apelin/apelin receptor (APLNR)-mediated Forkhead box O1 (FOXO1) suppression in ECs limits excessive FA transport and tissue accumulation, contributing to atheroprotecton against vascular diseases such as atherosclerosis [86–88]. Pharmacological inhibition of FABP4 with compounds such as BMS309403 enhances eNOS expression and improves EC function, reducing plasma triglyceride (TG) levels and atherosclerosis progression in diabetic models [89, 90]. In the context of aging, senescent ECs show increased expression of FA transporters, such as CD36 and FABP, suggesting a metabolic shift from glycolysis to FA oxidation to support a proinflammatory phenotype [91]. These findings highlight how age-related shifts in FA transporter expression contribute to EC dysfunction.

#### FA storage and lipolysis

Once inside a cell, FAs are esterified into TGs and stored within LDs, dynamic organelles derived from the ER. The biosynthesis and turnover of LDs are regulated by enzymes such as diacylglycerol O-acyltransferase (DGAT) and adipose TG lipase (ATGL) [92]. Moreover, autophagy components are involved in LD biogenesis. During prolonged starvation, autophagy breaks down membrane organelles to release FAs, which are re-esterified and sequestered into newly formed LDs for energy storage. Conversely, when cellular energy demands increase, LDs can be selectively degraded through autophagy-dependent lipophagy to release FAs for beta-oxidation and ATP production [93, 94].

In vascular ECs, LD accumulation is influenced by FA availability and DGAT1 and ATGL activities. For example, treatment with oleic acid promotes LD formation and triggers compensatory lipolysis through ATGL. Inhibition of ATGL delays FA release from LDs. LD-derived FAs reduce saturated FA-induced ER stress and enhance FAO, thereby reducing glycolysis to meet metabolic demands under normal conditions [95]. Although transient LD accumulation may serve as a protective buffer, chronic LD accumulation in ECs leads to decreased NO production, increased NF-κB signaling, elevated blood pressure, and accelerated atherosclerosis, particularly in ATGL-deficient or high-fat diet-fed mice [96, 97]. Inhibition of LD formation using a DGAT inhibitor restores NO production in ATGL-deficient mice [98]. Moreover, loss of ATGL1 triggers TG accumulation and ER stress-induced inflammation in ECs, contributing to plaque formation [99]. A high-fat diet induces LD accumulation, which impairs EC ciliation through stearoyl-CoA desaturase 1 (SCD1), promoting atherosclerosis [100].

While LDs are increasingly recognized as aging-associated features, their precise role in human vascular aging is not fully defined. Aged macrophages with dysfunctional autophagy exhibit increased LDs and lose their ability to regress atherosclerosis [101, 102]. In senescent ECs, increased LD accumulation was linked to elevated expressions of proinflammatory cytokines [91]. These findings highlight the impacts of neutral lipid and LD accumulation in ECs, suggesting that disrupted LD dynamics in ECs may contribute to vascular pathologies.

#### Fatty acid oxidation (FAO)

FAO is a mitochondrial process where FAs are converted into acetyl-CoA to provide energy for the TCA cycle and produce ATP. Although ECs primarily rely on glycolysis for ATP production, quiescent ECs exhibit increased FAO to support mitochondrial respiration and maintain a redox balance by producing NADPH [103]. In addition, senescent ECs display heterogenous metabolic reprogramming. While some studies reported increased glycolytic activity, lactate production, and enhanced TCA cycle activity and mitochondrial respiration during senescence [32], others showed a marked shift toward FAO and a reduced glycolytic rate and lactate production in senescent ECs compared to young ECs [91]. This metabolic shift may vary by cell type and mode of senescence induction [104]. Moreover, this metabolic shift from glycolysis to FAO in senescent ECs is closely associated with increased secretion of proinflammatory factors characteristic of the senescence-associated secretory phenotype (SASP), which exacerbates chronic vascular inflammation (often referred to as inflammaging) and contributes to vascular diseases such as atherosclerosis and hypertension [105]. Pharmacologic inhibition of carnitine palmitoyl transferase (CPT)1, the rate-limiting enzyme in FAO, using etomoxir attenuated SASP-related cytokine expression, suggesting a causal relationship between excessive FAO and EC inflammation during aging [3, 91]. FAO also plays a role in deoxynucleotide triphosphate (dNTP) synthesis and EC proliferation; silencing CPT1a decreases EC proliferation and metabolic homeostasis [106]. The role of increased FAO during senescence in promoting pathological angiogenesis remains unclear and warrants further investigation. Given the complexity of FAO regulation, which is influenced by the FA type, cellular environment, and coexisting risk factors, its contribution to atherosclerosis and therapeutic potential should be explored in greater depth.

#### De novo FA synthesis

De novo FA synthesis (de novo lipogenesis (DNL)) is vital for EC homeostasis, by regulating membrane formation, lipid signaling, and metabolic balance. ECs express key enzymes for DNL, including ATP citrate lyase (ACLY), acetyl-CoA carboxylase (ACC), and FA synthase (FASN), which convert citrate into long-chain FAs (LCFAs) such as palmitate [107]. With aging and metabolic disorders such as diabetes, DNL is disrupted. FASN expression decreases in EC-enriched tissues of diabetic mice, whereas insulin restores its levels and eNOS activity, highlighting the importance of DNL in glucose-sensitive endothelial regulation. In addition, FASN inactivation results in impaired eNOS palmitoylation, increased vascular permeability and inflammation, and defective angiogenesis, further reinforcing its regulatory role in EC health [108]. Aging and metabolic stress disrupt DNL in ECs, with senescent cells exhibiting decreased FASN expression, elevated inflammation, and loss of endothelial function [91]. Conversely, higher FASN expression may lead to pathological angiogenesis through malonyl-CoA-mediated inhibition of mammalian target of rapamycin (mTOR) complex 1 (mTORC) signaling [109]. This paradox underscores the complexity of FASN’s role in EC biology, suggesting that both excessive and insufficient DNL can contribute to endothelial dysfunction, depending on the context and temporal dynamics.

Sphingolipid metabolism is a closely related lipid pathway that produces ceramide and sphingosine-1-phosphate (S1P), which have contrasting effects (beneficial *vs*. detrimental) in many cellular processes. Aging skews the ceramide/S1P ratio in favor of ceramide accumulation, leading to increased oxidative stress, apoptosis, and impaired NO bioavailability [110]. The imbalance, known as the sphingolipid rheostat, contributes to atherosclerosis progression and plaque instability [111]. Interestingly, in a mouse model of coronary atherosclerosis, hemodynamic stress was shown to induce changes in sphingolipid metabolism that favored the production of S1P over ceramides, suggesting that blood vessels may be adaptative to stress [112].

In summary, aging-induced alterations in FA metabolism may disrupt EC function by reducing NO bioavailability, increasing oxidative stress, and promoting chronic inflammation. These metabolic disturbances contribute to the development of atherosclerosis. The precise mechanisms linking age-related FA metabolic reprogramming to EC dysfunction remain to be elucidated by further investigations. Understanding and targeting FA metabolic pathways in ECs may provide new therapeutic strategies for CVDs.

### Amino acid metabolism

**Fig. 4:**
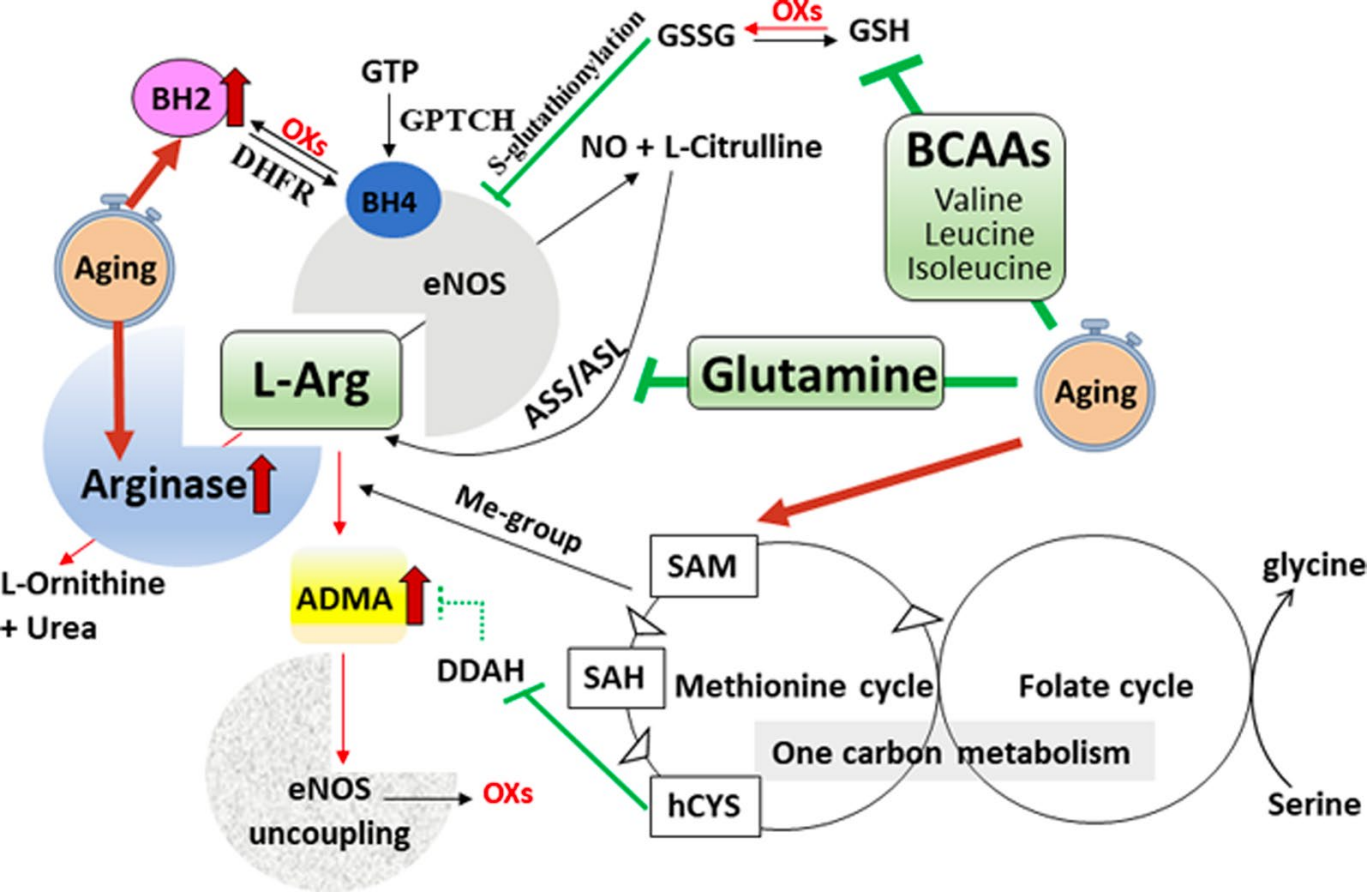
Dysregulation of amino acid metabolism and endothelial nitric oxide synthase (eNOS) uncoupling in the aging endothelium. This diagram illustrates how aging orchestrates key alterations in amino acid metabolism, ultimately leading to eNOS uncoupling and impaired nitric oxide (NO) production. The central pathway depicts l-Arginine as the substrate for eNOS to generate NO. However, aging drives several detrimental changes: it increases arginase activity, which competes with eNOS for l-Arginine; it promotes the accumulation of asymmetric dimethylarginine (ADMA), an endogenous eNOS inhibitor that directly impedes its function; and through oxidative stress (OXs), it causes the oxidation of essential cofactor tetrahydrobiopterin (BH4) to dihydrobiopterin (BH2), further impairing eNOS coupling. Furthermore, the accumulation of Glutamine and Branched-Chain Amino Acids (BCAAs), aggravated by aging, also contributes to eNOS uncoupling. In this figure, red arrows and text predominantly indicate processes or molecules that are increased or upregulated with aging or under conditions of oxidative stress, often culminating in eNOS uncoupling and reduced NO production. Conversely, green inhibitory lines represent detrimental inhibitory effects caused by aging, ultimately leading to harmful changes that result in eNOS uncoupling. The overall interplay within this metabolic network demonstrates how aging-induced metabolic shifts exacerbate oxidative stress, leading to endothelial dysfunction primarily through eNOS uncoupling. *GPTCH* guanosine triphosphate cyclohydrolase, *DHFR* dihydrofolate reductase, *GSH* glutathione, *GSSG* glutathione disulfide, *SAM* S-adenosylmethionine, *SAH* S-asenosylhomocysteine, *hCYS* homocysteine, *DDAH* dimethylarginine dimethylaminohydrolase, *Me-group* methyl-group

Metabolism of amino acids is critical for EC function, providing building blocks for proteins and supporting various metabolic processes. In senescent ECs, disruption of key amino acid pathways, particularly l-arginine (l-Arg), glutamate, and branched-chain amino acids (BCAAs), contributes to vascular dysfunction and atherosclerosis (Fig. 4) [19, 113, 114].

#### l-Arg

l-Arg is a conditionally essential amino acid involved in NO production, the urea cycle, and synthesis of multiple biomolecules [115, 116]. In ECs, l-Arg is converted to NO and L-citrulline by eNOS, a process crucial for vascular health [117]. However, in senescent ECs, eNOS activity declines, leading to impaired NO production and dysfunction [9, 118, 119]. The recycling of citrulline back to l-Arg via ASS/ASL (the citrulline-NO cycle) pathway sustains NO production even under low plasma l-Arg availability [120]. In addition to NOS, l-Arg can be catalyzed by arginase to produce urea and l-ornithine [117]. In aged or atherosclerosis-prone vasculature, arginase is upregulated, competing with eNOS for l-Arg, further reducing NO production and promoting EC dysfunction and atherosclerosis [121, 122]. Thus, arginase is emerging as a therapeutic target in vascular aging and atherosclerosis [123, 124].

In addition to NO synthesis, l-Arg can also be catalyzed by arginine decarboxylase to produce agmatine [117], which improves EC function by enhancing eNOS activity, reducing mitochondrial ROS levels, and alleviating lipid accumulation through the AMPK/phosphatidylinositol 3-kinase (PI3K)/Akt/eNOS signaling pathway [125, 126]. Meanwhile, posttranscriptional methylation of l-Arg residues in proteins generates asymmetric dimethylarginine (ADMA), a metabolic l-Arg analog byproduct and a natural NOS inhibitor that competes with l-Arg for binding, leading to NOS uncoupling (Fig. 4), EC dysfunction, and ultimately CVDs [127]. Elevated ADMA levels inhibit NO production, promote NOS coupling, and drive oxidative stress and vascular inflammation. Oxidative stress further enhances ADMA accumulation by suppressing dimethylarginine dimethylaminohydrolase (DDAH, ADMA hydrolase) activity, exacerbating EC dysfunction [128, 129]**.** Moreover, elevated plasma ADMA levels are a recognized hallmark of atherosclerosis [130]. NOS uncoupling is a pathological state in which NOS shifts from producing NO to generating superoxide (O₂⁻), an ROS. This shift contributes to oxidative stress, EC dysfunction, and atherosclerosis progression, particularly under cardiovascular risk factors such as diabetes, hypertension, dyslipidemia, smoking, and aging [131, 132]. ADMA-induced NOS uncoupling is closely linked to one-carbon metabolism, where S-adenosylmethionine (SAM) donates a methyl group for ADMA synthesis and is converted into S-asenosylhomocysteine (SAH), an inhibitor of methyltransferase [133]. SAH is then hydrolyzed to homocysteine, which suppresses DDAH activity, leading to ADMA accumulation [134]. A decreased SAM/SAH ratio, reflecting impairment, is associated with an increased risk of atherosclerosis in the elderly and may serve as a diagnostic biomarker [135, 136].

Moreover, tetrahydrobiopterin (BH_4_), a crucial eNOS cofactor, is oxidized to dihydrobiopterin (BH_2_) under oxidative stress, leading to eNOS coupling [131]. The cellular ratio of BH_4_/BH_2_ affects eNOS uncoupling and is implicated in the pathophysiology of EC dysfunction [137]. In senescent ECs, an elevated BH_2_/BH_4_ ratio exacerbates oxidative stress and inflammation [138]. Strategies targeting BH_4_ regeneration via guanosine triphosphate cyclohydrolase (GPTCH) or the BH_2_-reducing enzyme, dihydrofolate reductase (DHFR), may help preserve eNOS function [139, 140]. Notably, a BH_4_ deficiency lowers the glutathione (GSH)/glutathione disulfide (GSSG) redox ratio, promotes eNOS S-glutathionylation, and further impairs NO production, thereby contributing to endothelial dysfunction and vascular inflammation with aging [141, 142]. Disruptions of both BH_4_ availability and the GSH/GSSG balance are key mechanisms driving eNOS uncoupling during vascular aging.

Collectively, dysregulated l-Arg metabolism is the underlying cause of NO deficiencies, oxidative stress, and vascular aging. Although l-Arg supplementation has shown mixed results in clinical studies [113], maintaining a balanced l-Arg/NO pathway remain a promising therapeutic focus.

#### Glutamine

Glutamine is the most abundant non-essential aa in circulation, providing up to 70% of the TCA cycle’s carbon for ECs, a contribution comparable to that of glucose [143]. ECs metabolize glutamine via glutaminase 1 (GLS1) and glutamate dehydrogenase (GLUD) to α-ketoglutarate, which supports anabolic processes including nucleotide, protein, polyamine, glucosamine, and de novo serine synthesis [144]. Disruptions in glutamine-glutamate homeostasis are associated with CVDs [145]. Moreover, glutamine metabolism impacts other critical EC functions by inhibiting l-Arg regeneration from L-citrulline [73, 146, 147]. Notably, increased glutamine metabolism also supports the inflammatory state of macrophages within atherosclerotic lesions [148]. These findings suggest that age-related alterations in EC glutamine metabolism could contribute to EC dysfunction and atherosclerosis progression by affecting EC proliferation, the redox balance, and inflammatory responses. However, the specific mechanisms underlying these responses remain to be investigated.

Pyroglutamic acid (PCA), also known as 5-oxoproline, is a cyclic derivative of glutamic acid (or glutamine) and a gamma-glutamyl cycle metabolite indicative of glutathione turnover [149]. While glutamine’s role in atherosclerosis is being actively investigated, direct research specifically focusing on PCA’s independent role in the pathogenesis of atherosclerosis is limited. In this context, its importance seems to be more related to its involvement in the glutathione synthesis pathway and as a marker of altered glutamine/glutamate metabolism.

#### BCAAs

The three BCAAs of valine, leucine, and isoleucine are catabolized by BCAA aminotransferase (BCAT) and branched-chain α-ketoacid dehydrogenase (BCKD) enzymes into intermediates like acetyl-CoA and methylmalonay-CoA, which enter the TCA cycle [113, 150]. Age-related metabolic disorders such as hyperglycemia and hyperlipidemia elevate plasma BCAA levels, thereby accelerating atherosclerosis [151]. In senescent cells, this BCAA accumulation may be due to upregulated BCAA transporters (SLC6A14/15) or reduced BCAT/BCKD activity, which compromises antioxidant defenses by impairing glutathione synthesis and enhances oxidative stress and inflammation via mTORC and NF-κB signaling pathways [152–155]. While elevated systemic BCAA levels were correlated with increased atherosclerosis risks, the direct role of BCAA metabolism within ECs during atherogenesis, particularly with aging, remains unclear. There is evidence that BCAAs can promote EC dysfunction by increasing oxidative stress and inflammation, but the specific enzymes, transporters, and pathways involved as well as the context-dependent effects require further investigation to fully elucidate their contribution to atherosclerosis development.

### Mitochondrial respiration

Mitochondrial respiration encompasses oxidative phosphorylation (OXPHOS) and the TCA cycle**.** It plays a central role in cellular bioenergetics, providing ATP through OXPHOS and supporting macromolecular biosynthesis, including nucleotides, lipids, heme, and iron-sulfur clusters [156]. The TCA cycle serves as a metabolic hub that integrates substrates such as FAs, aas, and pyruvate to generate NADH and flavin adenine dinucleotide (FADH_2_), which then provide energy for the electron transport chain to produce ATP [157]. In addition to energy regulation, TCA intermediates, including acetyl-CoA and citrate**,** modulate innate and adaptive immune responses and influence gene expressions via histone acetylation [157, 158].

Acetyl-CoA can be derived from various sources, including pyruvate, FAO, aa degradation, and acetate. Metabolic enzymes, such as acetyl-CoA synthetase short-chain family member-1 (ACSS), facilitate acetate’s conversion into acetyl-CoA [158]. Under hyperglycolytic conditions, abnormal acetate production increases acetyl-CoA levels through ACSS2, promoting TGF-β/Smad2/4 acetylation and the EndoMT, which contribute to the development of atherosclerosis [60].

Although mitochondria are the major ATP producers in most cells, ECs preferentially rely on glycolysis, generating only ~ 15% of their ATP via OXPHOS [23, 29]. However, ECs maintain a substantial spare mitochondrial respiratory capacity, which can be mobilized during metabolic stress, such as glucose deprivation, by utilizing FAs and glutamine [27]. Thus, mitochondria in ECs may primarily serve as signaling hubs rather than energy generators [29]. Although mitochondrial respiration supports angiogenesis, its alternations in atherogenesis remain underexplored [159].

Mitochondrial respiration is the primary source of cellular ATP. However, its electron transport chain can physiologically generate ROS essential for regulating cell signaling, growth, and differentiation [160]. With aging, ROS overproduction triggers oxidative stress, directly impairing mitochondrial functions in ECs. This impairment manifests as reduced mitochondrial biogenesis, dysregulated mitochondrial dynamics (imbalances in fusion and fission), and inefficient mitophagy, collectively accelerating EC dysfunction, senescence, and atherosclerotic progression [161–164]. Excessive ROS directly damage mitochondrial components (mitochondrial (mt)DNA, proteins, and lipids), further inhibiting mitochondrial biogenesis [165, 166]. Moreover, this oxidative stress often diminishes AMP-activated protein kinase (AMPK) activity, which then reduces the expression of PPARγ coactivator (PGC)-1α, a pivotal regulator of mitochondrial biogenesis, thus exacerbating the decline in the mitochondrial mass [167]. Beyond the change in mass, ROS are a key trigger of mitochondrial fragmentation (favoring fission over fusion) via dynamin-related protein 1 (DRP1) activation [168–170]. While fragmentation aids in tagging mitochondria for mitophagy, if the mitophagy pathway (e.g., phosphatase and tensin homolog (PTEN)-induced kinase 1 (PINK1)/Parkin) is compromised by age-related factors or the sheer volume of damaged mitochondria, these fragmented, dysfunctional mitochondria accumulate instead of being cleared [171]. This accumulation further exacerbates mitochondrial ROS production, creating a vicious cycle that accelerates cellular aging and contributes to various age-related pathologies, including CVDs [161, 172].

**Fig. 5:**
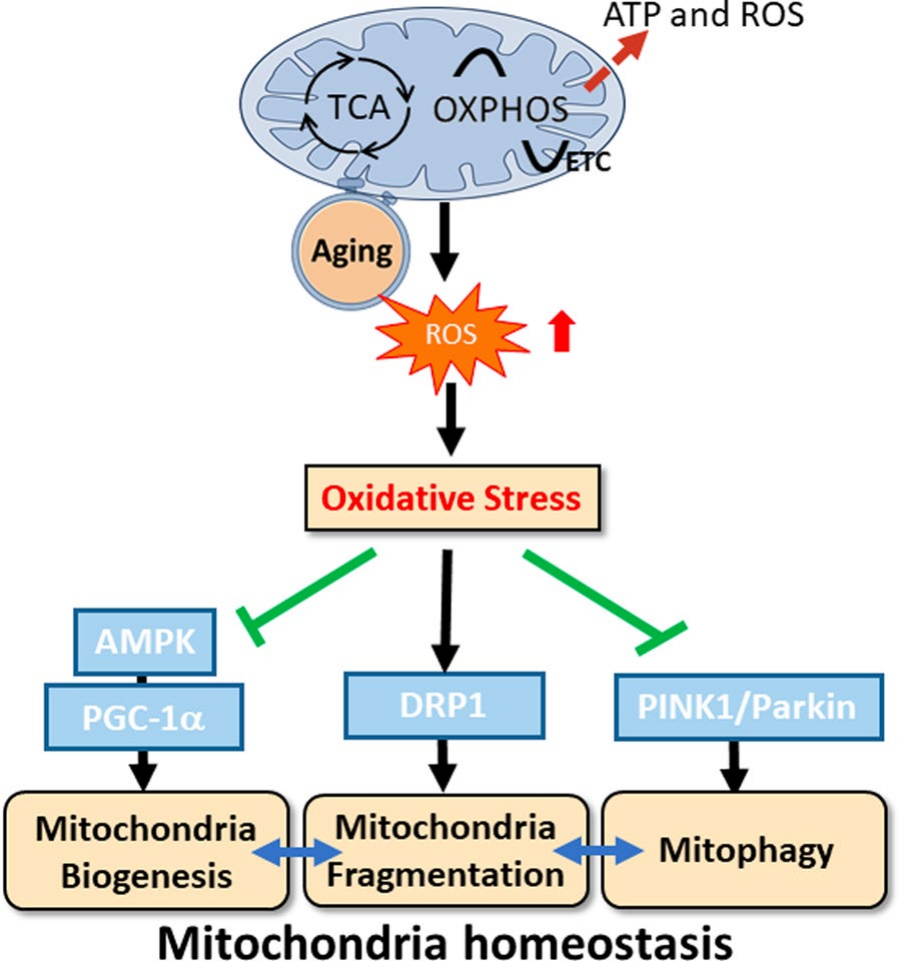
Aging-induced mitochondrial dysfunction via reactive oxygen species (ROS) overproduction. This figure illustrates how aging directly impacts mitochondrial function, leading to dysregulated mitochondrial respiration (TCA cycle and OXPHOS) and an overproduction of reactive oxygen species (ROS). The escalating ROS levels promote oxidative stress, which broadly impairs mitochondrial dynamics and quality control. Specifically, oxidative stress exerts inhibitory effects on crucial processes: it suppresses mitochondria biogenesis by inhibiting the AMPK/PGC-1α pathway, and it contributes to mitochondria fragmentation through its influence on dynamin-related protein 1 (DRP1). Furthermore, oxidative stress potentially impairs mitophagy, a vital quality control process regulated by proteins like PINK1/Parkin.

Taken together, mitochondrial respiration is tightly linked to EC metabolism and vascular health. Aging-induced mitochondrial dysfunction plays a pivotal role in promoting EC dysfunction and atherosclerosis (Fig. 5). Future therapeutic strategies aimed at restoring mitochondrial homeostasis may hold promise for reducing vascular aging.

### Aging-related circulating metabolites regulate EC functions

Arachidonic acid (AA), a membrane phospholipid component, is metabolized by cyclooxygenases (COXs), lipoxygenases (LOXs), and cytochrome P450 enzymes to generate eicosanoids including prostaglandins (PGs), thromboxanes (TXs), leukotrienes, and epoxyeicosatrienoic acids (EETs) [173, 174]. These metabolites regulate vascular tone, platelet aggregation, and inflammation. Healthy ECs predominantly produce prostacyclin (PGI2) via COXs, promoting vasodilation and inhibiting thrombosis, whereas thromboxane A2 (TxA2) exerts opposing effects [175]. Aging disrupts this balance, reducing the production of vasodilatory and anti-inflammatory eicosanoids while increasing vasoconstrictive and proinflammatory mediators, thereby exacerbating endothelial dysfunction and cardiovascular risks [176]. Notably, AA metabolites exhibit dual effects depending on the concentration and enzymatic context, contributing to vascular inflammation to varying degrees.

Carnosine, a dipeptide synthesized from β-alanine and L-histidine, plays a multifaceted role in attenuating age-related EC dysfunction and atherosclerosis through antioxidant, anti-inflammatory, and aldehyde-scavenging mechanisms [177, 178]. Aging reduces carnosine levels due to decreased carnosine synthase 1 (CARNS1) and increased carnosinase 2 (CNDP2) activity, impairing its protective capacity in ECs [179]. β-Alanine supplementation elevates carnosine levels, which decline with aging, offering potential therapeutic benefits for cardiovascular health [180]. While animal and in vitro studies have highlighted carnosine’s direct anti-atherogenic effects, human trials primarily demonstrated β-alanine’s role in boosting exercise tolerance, which may reduce cardiovascular risk [181, 182]. Combined supplementation strategies (e.g., β-alanine with antioxidants) could enhance EC protection, although clinical evidence in humans remains limited [183].

5-Methoxytryptophan (5-MTP), an EC-derived metabolite synthesized via tryptophan hydroxylase-1 (TPH-1) and hydroxyindole O-methyltransferase, has emerged as having a critical vasoprotective role by suppressing vascular inflammation and maintaining endothelial function. Age-associated proinflammatory stimuli, such as lipopolysaccharide (LPS) and TNF-α, downregulate tryptophan hydroxylase (TPH)-1, reducing 5-MTP production and contributing to EC dysfunction [184, 185]. Physiologically, 5-MTP supports vascular homeostasis by preserving VE-cadherin integrity via inhibiting p38 MAPK signaling and sustaining vascular endothelial growth factor receptor 2 (VEGFR2) activation, thus promoting EC proliferation and migration [185]. Exogenous 5-MTP improves endothelial repair, reduces vascular permeability, and prevents intimal hyperplasia in aging models [186]. Moreover, its effects are not limited to ECs, but it also regulates vascular smooth muscle cells and macrophage activation, suggesting that it may coordinate a defense against age-related vascular dysfunction. However, future studies are need to explore upstream regulatory mechanisms that may restore 5-MTP levels in aging ECs, such as nutritional interventions, transcriptional control of TPH-1, or modulation of enzyme activity involved in 5-MTP biosynthesis.

**Fig. 6:**
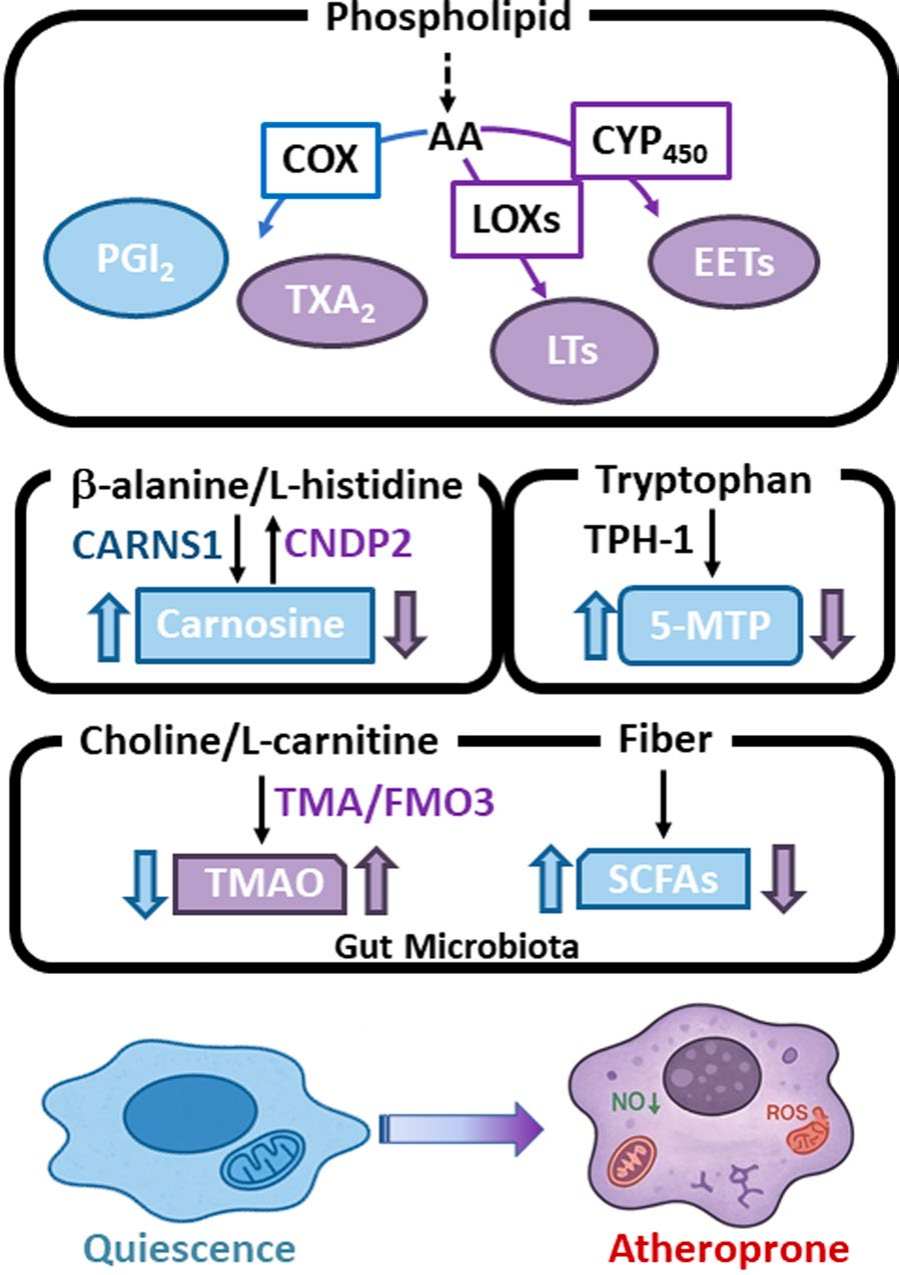
Aging-related circulating and microbial metabolites that regulate EC metabolism and function. This figure highlights the roles of key circulating and microbial-derived metabolites—such as arachidonic acid (AA) metabolites, carnosine, 5-methoxytryptophan (5-MTP), trimethylamine N-oxide (TMAO), and short-chain fatty acids (SCFAs)—in modulating endothelial inflammation, oxidative stress, and metabolic adaptation. Age-related declines in protective metabolites and increases in pro-atherogenic compounds contribute to EC dysfunction. *COXs* cyclooxygenases, *LOXs* lipoxygenases, *CYP450* cytochrome P450, *PGI*_*2*_ prostacyclin I_2_, *TXA*_*2*_ thromboxane A_2_, *LTs* leukotrienes, *EETs* epoxyeicosatrienoic acids, *CARNS1* carnosine synthase 1, *CNDP2* carnosinase 2; *TPH1* tryptophan hydroxylase-1, *TMA* trimethylamine, *FMO3* flavin-containing monooxygenase 3

Emerging evidence has connected gut microbiota-derived metabolites to endothelial dysfunction and atherosclerosis, particularly in aging populations. Key microbial metabolites, including trimethylamine N-oxide (TMAO) and short-chain FAs (SCFAs), directly influence EC function [187]. TMAO, which is produced from dietary choline and L-carnitine (found in red meat) via microbial trimethylamine (TMA) and hepatic flavin-containing monooxygenase 3 (FMO3), increases with age and contributes to oxidative stress, EC dysfunction, and plaque instability [188, 189]. In contrast, SCFAs (e.g., butyrate and propionate), which are derived from microbial fermentation of dietary fiber, exert protective effects by reducing inflammation and supporting EC integrity [190, 191]. However, microbial diversity and the production of these beneficial SCFAs, particularly propionate and butyrate, significantly decline in elderly individuals, potentially diminishing their vascular protection [190]. Taken together, these finding suggest that age-related gut dysbiosis exacerbates EC dysfunction and atherogenesis. Targeting the gut microbiotic composition through diet or other interventions may offer novel strategies for protecting endothelial health during aging (Fig. 6).

## Shear stress-regulated metabolic reprogramming of ecs in aging and atherosclerosis

ECs are constantly exposed to blood flow-derived shear stress [192], which critically determines their metabolic phenotype and function. LS or pulsatile shear stress (PS) maintains EC quiescence and anti-inflammatory properties, whereas OS, often occurring at arterial bifurcations and curvatures, induces EC activation, oxidative stress, and inflammation, thereby contributing to atherosclerotic lesion formation [6, 193]. Exercise-induced shear stress enhances EC function and vascular remodeling [194]. Notably, exercise training improves EC-dependent vasodilation in elderly patients with CAD, in part by activating eNOS [195]. Under LS, ECs align with the flow, remain quiescent, and produce high levels of NO. In contrast, OS that occurs at arterial curvatures and branches increases inflammation, oxidative stress, and apoptosis while reducing NO, promoting a pro-atherogenic EC phenotype [6, 196–198]. Aging-associated arterial stiffening further disrupts flow patterns, potentially exacerbating OS and promoting EC dysfunction [11, 12, 193, 199]**.**

### Atheroprotective effects of LS and their decline with aging

LS promotes EC quiescence by inducing Krüppel-like factors (KLFs), which are associated with age-related health and longevity. KLFs, particularly KLF2 and KLF4, are critical shear-responsive transcription factors that maintain the vascular barrier and endothelial quiescence by promoting anti-inflammatory and anti-thrombotic phenotypes and regulating metabolism [200, 201]. However, the precise mechanism by which KLF2/4 regulates EC metabolism in atherosclerosis progression remains unclear. Notably, atheroprotective LS activates KLF2 expression in ECs, whereas disturbed flow or OS reduces its expression through upregulating miR-92a [202]. KLF2 suppresses glycolysis by downregulating the glycolytic enzymes 6-phosphofructo-2-kinase (PFKFB3), hexokinase 2 (HK2), and phosphofructokinase 1 (PFK1), thereby inhibiting EC angiogenic activity [203]. KLF4 also inhibits glycolysis under PS/LS compared to OS through glucokinase regulator protein (GCKR) transcription and AMPK-dependent GCKR phosphorylation, as demonstrated in a high-level voluntary running mouse model of exercise training [204]. This metabolic shift in LS-mediating glycolysis reduces eNOS O-GlcNAcylation, thereby enhancing eNOS phosphorylation and NO production [205]. Using a combination of transcriptomics and tracer metabolomics, Simões-Faria et al. meticulously mapped the metabolic adaptation of ECs to LS, revealing a dramatic metabolic shift: a reduction in glycolytic flux and a clear preference for glutamine as the dominant carbon substrate fueling central metabolic pathways [206]. In addition to its role in glycolysis, LS also promotes mitochondrial biogenesis, which is crucial for tissue health. The LS enhances mitochondrial respiration function and decreases glycolysis and lactate production in vitro and in an exercised mice model [207, 208]. These benefits in mitochondrial biogenesis and function are mediated by KLF4 and NAD^+^-deacetylase sirtuin 1 (SIRT1), which maintain mitochondrial homeostasis via estrogen-related receptor (ERR)/PGC-1 and oxidative stress resistance [209–213]. Specifically, KLF expression by ECs markedly declines with age, contributing to reduced eNOS levels [214, 215]. Thus, the age-related decline of KLF2/4 compromises shear stress-mediated metabolic adaptations and contributes to endothelial dysfunction.

As a key energy sensor in ECs, AMPK regulates glycolysis, mitochondrial function, autophagy, and lipid metabolism, contributing to vascular homeostasis and stress defense. The decline in AMPK signaling with age impairs metabolic control [216]. Paradoxically, while LS typically inhibits glycolysis by GCKR phosphorylation and downregulation of glycolytic enzymes (PFKFB3, HK2, and PFK1) [204], Cronin et al. found that LS can also enhance AMPK activity and glucose uptake, stabilizing the actin cytoskeleton and aligning cells [217]. AMPK also modulates FA metabolism by reducing hydroxy methylglutaryl coenzyme A reductase (HCR) activity, a rate-limiting enzyme for cholesterol synthesis, via phosphorylation and degrading FOXO1 in ECs upon LS stimuli [218]. This paradoxical nature extends to OS, which increases PRKAA1/AMPKα1 to induce glycolysis; yet its absence in ECs exacerbates atherosclerosis [41]. These contrasting effects of LS and OS underscore the context-dependent dual role of AMPK in metabolic regulation and imply a protective function of PRKAA1/AMPKα1 against atherosclerosis.

These age-related disruptions in AMPK signaling may explain the diminished protective effects of LS on endothelial metabolism in elderly people. Pharmacologic activation of AMPK (e.g., by metformin or berberine) was shown to delay senescence and restore metabolic homeostasis, supporting its therapeutic potential in combating vascular aging and shear stress-related endothelial dysfunction [219].

### OS-induced glycolysis and inflammation are exacerbated by aging

Hypoxia-inducible factor (HIF)-1α is a critical transcription factor responsive to hypoxia and mechanical forces, notably shear stresses, and plays a central role in EC metabolic adaptation during atherosclerosis and aging [220, 221]. Under OS, HIF-1α is stabilized via nitrogen oxide (NOX)/ROS, which upregulates glycolytic enzymes (SLC2A1 and HK) and inhibits mitochondrial respiration via PDK1 activation [222]. OS also activates NF-κB and Cezanne to further promote HIF-1α transcription and stabilization, which in turn enhances the glycolytic capacity through upregulating glycolysis regulators and enzymes (PFKFB3, HK2, and enolase), driving EC proliferation and inflammation [221].

Recent studies also identified Yes-associated protein (YAP) and transcriptional coactivator with the PDZ-binding motif (TAZ) as key mechanoresponsive factors in the Hippo pathway to transmit blood flow-induced mechanical forces into intracellular signaling to affect EC phenotypes [223]. Specifically, OS activates YAP/TAZ to upregulate EC glycolysis via PFKFB3 induction, enhancing EC proliferation, inflammation, and neovascularization [35]. In contrast, LS inhibits YAP/TAZ to maintain EC quiescence [224–227]. These results suggest that different flow patterns, mediated by YAP/TAZ, transduce mechanical signals from blood flow into ECs and regulate glycolytic metabolism to influence atherogenesis.

**Fig. 7:**
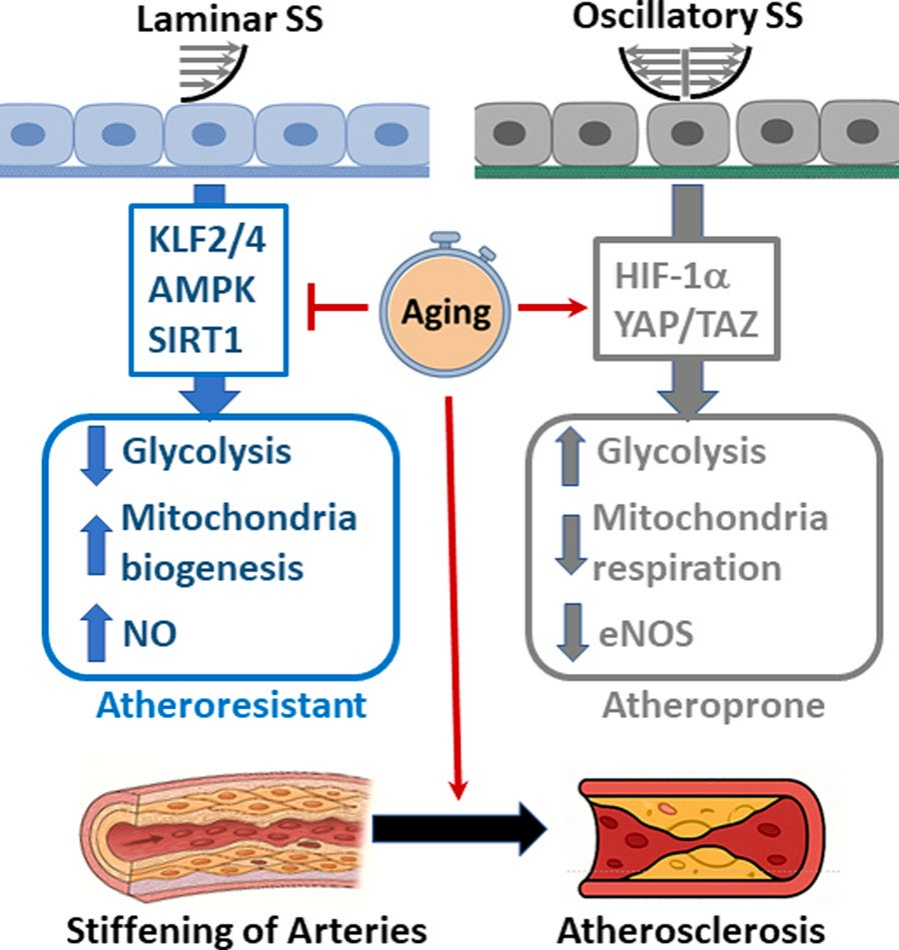
Shear stress-mediated mechanotransduction pathways in regulating EC metabolism and phenotype. Laminar shear stress induces protective signaling via KLF2/4, AMPK, and SIRT1 to promote mitochondrial biogenesis and NO production, whereas oscillatory shear stress activates HIF-1α and YAP/TAZ to enhance glycolysis and inflammation. Aging gradually affects the stiffening of arteries and disrupts these shear-responsive networks, by shifting the endothelium from an atheroprotective to an atheroprone state

Taken together, atheroprone OS and atheroprotective LS differentially modulate EC metabolism and function. OS reduces the activity of shear-responsive regulators (KLF2 and KLF4), promotes glycolysis, and inhibits mitochondrial respiration, contributing to EC dysfunction and atherosclerosis. LS decreases glycolysis and enhances mitochondrial biogenesis, preventing vascular damage. The intricate relationship between pathological functions and metabolism in ECs offers a compelling new research area for exploring how shear stresses influence endothelial phenotype and function. The shear stress medicated-mechanotransducers regulating these distinct effects are shown in Fig. 7.

### Other metabolic pathways regulated by shear stress

In addition to glycolysis, FA metabolism, and mitochondrial respiration, shear stress regulates other metabolic pathways in ECs with relevance to aging and vascular dysfunction. LS increases AA metabolism, which is a critical pathway in vascular tone and inflammation. LS was shown to increase AA release and prostaglandin I2 (PGI_2_) production in human ECs [228, 229]. Taba et al. reported that LS selectively upregulates lipocalin-type prostaglandin D synthase (L-PGDS), leading to elevated levels of prostaglandin D2 (PGD2) and its downstream anti-inflammatory metabolite, 15d-PGJ2, in the extracellular environment [230]. Given that AA-derived eicosanoids (prostaglandins, thromboxanes, and leukotrienes) play divergent roles in vasoconstriction, inflammation, and platelet aggregation [173], the roles of various LS-induced AA metabolites in modulating EC function and atherogenesis require further investigation.

An analysis of microarray data from ApoE⁻/⁻ mice with disturbed flow revealed that OS alters β-alanine metabolism, particularly via epigenetic mechanisms [231, 232]. β-Alanine, a component of the antioxidant dipeptide carnosine, is synthesized via aldehyde dehydrogenases ALDH2 and ALDH3A1 [175]. Zhao et al. demonstrated that OS triggers DNA methyltransferase 1 (DNMT1)-dependent methylation of ALDH promoters, resulting in decreased ALDH expression and β-alanine synthesis and reducing carnosine availability. This is accompanied by enhanced Smad2/3 phosphorylation to promote the EndMT and atherosclerosis [232]. These findings suggest that flow-mediated regulation of β-alanine metabolism plays a role in EC dysfunction and atherogenesis.

## Emerging approaches for deciphering endothelial metabolism under aging and shear stress

EC metabolism, particularly under aging and changes in flow patterns and associated shear stress, exhibits distinct characteristics that are insufficiently understood due to the limitations of traditional in vitro methods. These methods often overlook systemic and cellular heterogeneity crucial for capturing in vivo metabolic adaptations. Recent advances in single-cell metabolomics now enable label-free, high-resolution analysis of metabolites in individual ECs using mass spectrometry (MS). Manzo et al. employed fluorescence activated cell sorting (FACS) and a liquid chromatographic-tandem MS (LC/MS/MS) analysis of sphingolipid levels to show that coronary ECs in atherosclerosis undergo sphingolipid remodeling, increasing the S1P/ceramide ratio as a protective response to hemodynamic forces [112]. While single-cell metabolomics is a powerful tool for profiling individual cell metabolism in tissues, the technically demanding isolation and preparation of tissue samples for metabolic analysis without introducing artifacts or altering the native metabolic state remain significant challenges. MS imaging (MSI) has emerged as a complementary technique, enabling spatial resolution of cell metabolism in tissues. A study by Sun et al. used MSI to demonstrate, for the first time, spatially resolved reprogramming of carnitine metabolism and altered expression of carnitine palmitoyl transferase 2 (CPT2) and carnitine acetyltransferase (CAT) in breast cancer [233]. However, technical limitations still hinder comprehensive coverage of single-cell metabolomes.

Integrating multi-omics data, a pathway analysis, and computational modeling may offer powerful tools to investigate metabolic reprogramming in ECs during aging and atherosclerosis, as exemplified by Kalucka et al.’s single-cell RNA-sequencing (scRNA-Seq) atlas of 11 mouse tissues, which revealed 78 EC subclusters and highlighted tissue origin as the primary determinant of EC heterogeneity, including tissue-specific metabolic profiles [234]. Similarly, Ma et al. combined scRNA-Seq and a metabolic flux analysis to identify glucose pathway shifts and intercellular interactions in human atherosclerotic plaque [235]. Zhao et al.’s analysis of Dunn et al.’s microarray data showed that shear stress significantly impacts metabolic pathways, particularly β-alanine metabolism, in ApoE^−/−^ mice [231, 232].

**Fig. 8:**
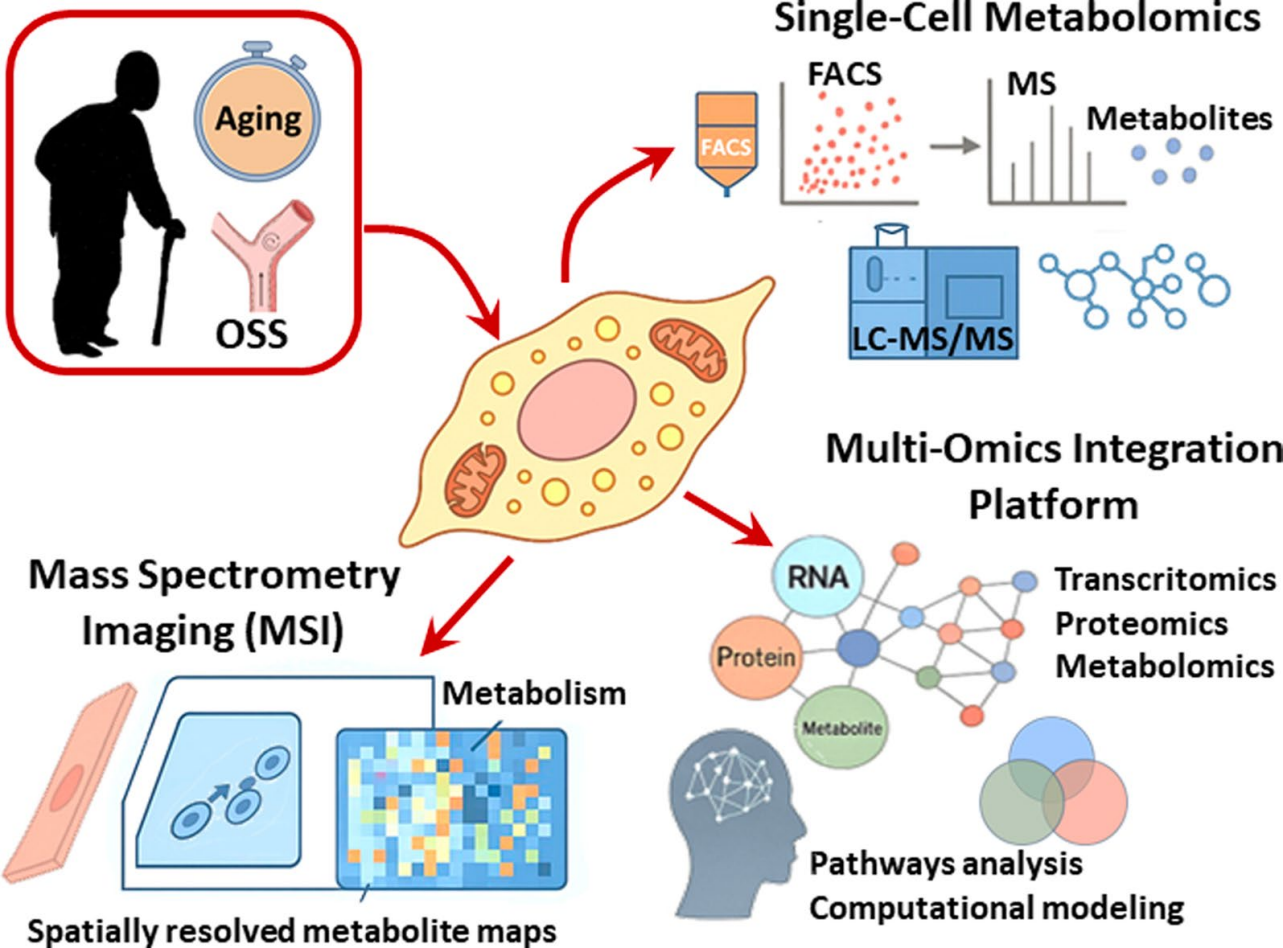
Emerging technologies for profiling endothelial metabolism under aging and shear stress. Single-cell metabolomics (via FACS and MS), mass spectrometric imaging (MSI), and multi-omics integration platforms (transcriptomics, proteomics, and metabolomics) enable high-resolution analysis of endothelial metabolic heterogeneity and spatial metabolic mapping. These tools are essential for decoding the metabolic reprogramming driving age-related vascular dysfunction and atherosclerosis.

In summary, single-cell metabolomics and integrative multiomics offer powerful tools for elucidating how aging and mechanical forces remodel EC metabolic reprogramming. By resolving cellular heterogeneity and integrating different layers of molecular information, these approaches can provide unprecedented insights into the mechanisms driving vascular dysfunction and identify novel therapeutic strategies (Fig. 8).

## Clinical and therapeutic implications of endothelial metabolic reprogramming in vascular aging and atherosclerosis

Given the significant roles of metabolites in atherosclerosis, targeting specific metabolic pathways in ECs will be developed as a novel therapeutic strategy. Prior to treatment, a reliable method for plaque tracking and monitoring drug therapy responses is required. Aging blood vessels accumulate disturbed shear stresses, oxidized low-density lipoprotein (ox-LDL), and inflammatory cytokines, which trigger EC metabolic reprogramming, notably an increase in glycolysis. [1, 3]. Therefore, glycolysis upregulation in aged and OS-exposed vessels may serve as a disease biomarker and therapeutic target for elevating EC dysfunctions and atherosclerosis. Fluorodeoxyglucose (FDG), a glucose analogue, is taken up by high-glucose-demanding cells. Once inside cells, FDG is phosphorylated by hexokinase (HK), but cannot be further metabolized, leading to intracellular accumulation detectable by imaging. The ^18^F-FDG tracer is commonly used to non-invasively image inflammatory cells (with high glycolysis rates) by positron emission tomography (PET) [236, 237]. It was also linked to increased FDG uptake with severe inflammation in cardiovascular atherosclerosis [238]. Thus ^18^F-FDG-PET can help assess the overall activity of atherosclerotic disease and monitor the response to therapies [239].

### Traditional atherosclerotic drugs in regulating EC metabolism

Traditional US Food and Drug Administration (FDA)-approved atherosclerosis treatments primarily target risk factors and prevent disease progression. Core treatments, including lipid-lowering medications, particularly statins (e.g., atorvastatin) which reduce LDL-cholesterol (LDL-C) and stabilize plaque, are foundational. Other lipid-lowering agents include proprotein covertase subtilisin/kexin type 9 (PCSK9) inhibitors (e.g., Inclisiran, Alirocumab, and Evolocumab) and ezetimibe. The PCSK9 inhibitor, Lerodalcibep, is currently under USFDA Biologics License Application (BLA) review (PDUFA date: Dec. 2025). Antiplatelet drugs like aspirin and clopidogrel prevent blood clots, while antihypertensives (e.g., angiotensin-converting enzyme (ACE) inhibitors and beta-blockers) offer indirect benefits by protecting blood vessels. Notably, diabetes drugs such as SGLT2 inhibitors (e.g., Empagliflozin, Dapagliflozin, and Canagliflozin) have also shown potential in slowing atherosclerosis [240]. Additionally, the anti-inflammatory agent, colchicine, and emerging nanoparticle-based systems offer improved vascular targeting with reduced systemic toxicity [241, 242]**.**

Several of these drugs exhibit direct or indirect effects on EC metabolism, which is increasingly recognized as central to vascular aging and atherosclerosis. Statins inhibit β-hydroxy β-methylgluraryl (HMG)-CoA reductase (HCR), thereby reducing cholesterol and isoprenoid synthesis. This downregulates small guanosine triphosphatase (GTPase) prenylation (e.g., RhoA activation), which otherwise suppresses eNOS expression and promotes EC inflammation [243–246]. Additionally, statins enhance NO bioavailability and decrease inflammation through regulating l-arginine and mevalonate metabolism [247–250]. However, statin-induced farnesyl pyrophosphate (FPP) reduction may impair mitochondrial coenzyme Q (CoQ10) production, with potential adverse effects on eNOS coupling and ATP production [251, 252]. Therefore, while statins have significant benefits, their effects on isoprenoids and mitochondrial function need to be careful considered. PCSK9 inhibitors, in addition to stabilizing LDL receptors (LDLRs) to lower LDL-C [253], also protect EC function by reducing inflammation and oxidative stress, thus attenuating plaque vulnerability [254]. However, the effects of PCSK9 in regulating EC metabolism and atherosclerosis still need to be investigated. Recognizing that aging and oscillatory flow stimulate EC glycolysis, SGLT2 inhibitors (e.g., empagliflozin) may provide metabolic benefits by blocking excessive glucose uptake under stress-induced SGLT2 expression [255]. This could help preserve EC homeostasis in aging-associated vascular dysfunction.

### Emerging therapeutic targets in EC metabolism

In addition to traditional drugs, targeting metabolic pathways altered by aging and shear stress offers new therapeutic opportunities. 6-Phosphofructo-2-kinase/fructose-2,6-biphosphatase (PFKFB3 is a key glycolytic regulator upregulated by disturbed flow, promoting EC proliferation, inflammation, and angiogenesis, while laminar shear stress suppresses its expression to maintain EC quiescence [35, 38, 42, 44, 56, 202, 220]. Inhibition of PFKFB3 with 3-(3-pyridinyl)-1-(4-pyridinyl)-2-propen-1-one (3PO) reduces pathological angiogenesis but faces clinical limitations due to solubility and selectivity issues [37, 255]. Its optimized 3PO derivative, PFK158, showed enhanced tolerability and reduced glycolytic flux in preclinical studies, was selected as the first-in-human, first-in-class PFKFB3 inhibitor, and has entered phase I clinical trials for advanced solid malignancies [256]. Notably, PFK158 significantly reduced glucose uptake and lactic acid production, and promoted atherosclerotic plaque stability [34], highlighting its anti-atherosclerosis potential.

Aging also increases oxidative stress due to mitochondrial dysfunction, impairing NO production and promoting endothelial dysfunction. Traditional antioxidants have limited efficacy due to poor targeting [257]. In contrast, mitochondrion-targeted antioxidants selectively eliminate ROS at the source, offering a promising strategy for vascular health. Mitoquinone (MitoQ), a meticulously engineered mitochondrion-targeting antioxidant, exerts its protective effects by selectively accumulating within the inner mitochondrial membrane, where it efficiently neutralizes ROS, thus safeguarding these vital organelles from oxidative damage [258, 259]. With a proven safety profile established through phase II clinical trials in Parkinson’s and liver disease [260, 261], MitoQ supplementation effectively rejuvenates vascular EC function in aged mice by restoring mitochondrial superoxide balance and enhancing NO bioavailability [262], a finding that was subsequently validated in a rigorous randomized, placebo-controlled, double-blind clinical trial involving healthy older adults [263]. While these compelling results strongly suggest MitoQ’s promise in combating age-related vascular dysfunction, its specific therapeutic potential in the complex landscape of atherosclerosis remains an area of active and crucial investigation.

In addition to blocking the metabolic pathway as drug development, some endogenous metabolites, such as carnosine and 5-MTP, have been used in vascular health and to prevent atherosclerosis progression, as mentioned above. Carnosine scavenges lipid peroxidation-derived aldehydes (e.g., 4-hydroxynonenal (HNE) and malondialdehyde (MDA)), reducing EC damage and inhibiting early atherosclerotic lesion formation in ApoE-null mice by 34% and reduced aldehyde-protein adducts in lesions [180]. Serum 5-MTP levels correlate with inflammatory states, serving as a theranostic marker for vascular dysfunction. 5-MTP’s dual action on ECs and smooth muscle cells positions it as a lead compound for therapies targeting age-related CVDs [184, 185]. While preclinical data are robust, human trials are needed to validate dosing and efficacy in aging populations.

### Therapeutic gut microbiota-derived metabolites

The emerging understanding of the gut microbiota’s role in atherosclerosis opens up new avenues for therapeutic interventions. Diets rich in fiber promote the growth of beneficial bacteria that produce short-chain fatty acids (SCFAs), which have anti-inflammatory and atheroprotective effects. Limiting the intake of choline, carnitine, and betaine can reduce the production of trimethylamine-N-oxide (TMAO), a pro-atherosclerotic metabolite [264]. Therapeutic strategies under development include TMAO-inhibiting agents that block microbial enzymatic conversion of dietary precursors [265], and short-term targeted antibiotic therapy might be used to selectively reduce harmful bacteria. However, due to risks like antibiotic resistance, these approaches must be carefully managed. Fecal microbiota transplantation (FMT) involves transferring fecal matter from a healthy donor to a recipient to restore a healthy gut microbiota. This approach has shown promise in treating various gut-related disorders and may have potential in atherosclerosis therapy. Other interventions include postbiotics, which utilize bacterial metabolites such as SCFAs without introducing live bacteria, offering a safer and more-controlled alternative. The gut microbiota’s impact on atherosclerosis varies between individuals, highlighting the need for further research and personalized strategies. Tailoring therapies to individual microbial profiles may enable more-effective prevention and treatment by targeting microbiota-derived metabolites. TMAO exerts its pro-atherogenic effects by increasing oxidative stress, promoting inflammation [266], and impairing NO signaling [189]. 3,3-Dimethyl-1-butanol (DMB) was shown in animal models to inhibit TMAO production and attenuate EC dysfunction, positioning it as a promising candidate for preventing vascular damage driven by microbial metabolites [267]. However, further research is necessary to translate these findings into clinical practice.

**Fig. 9:**
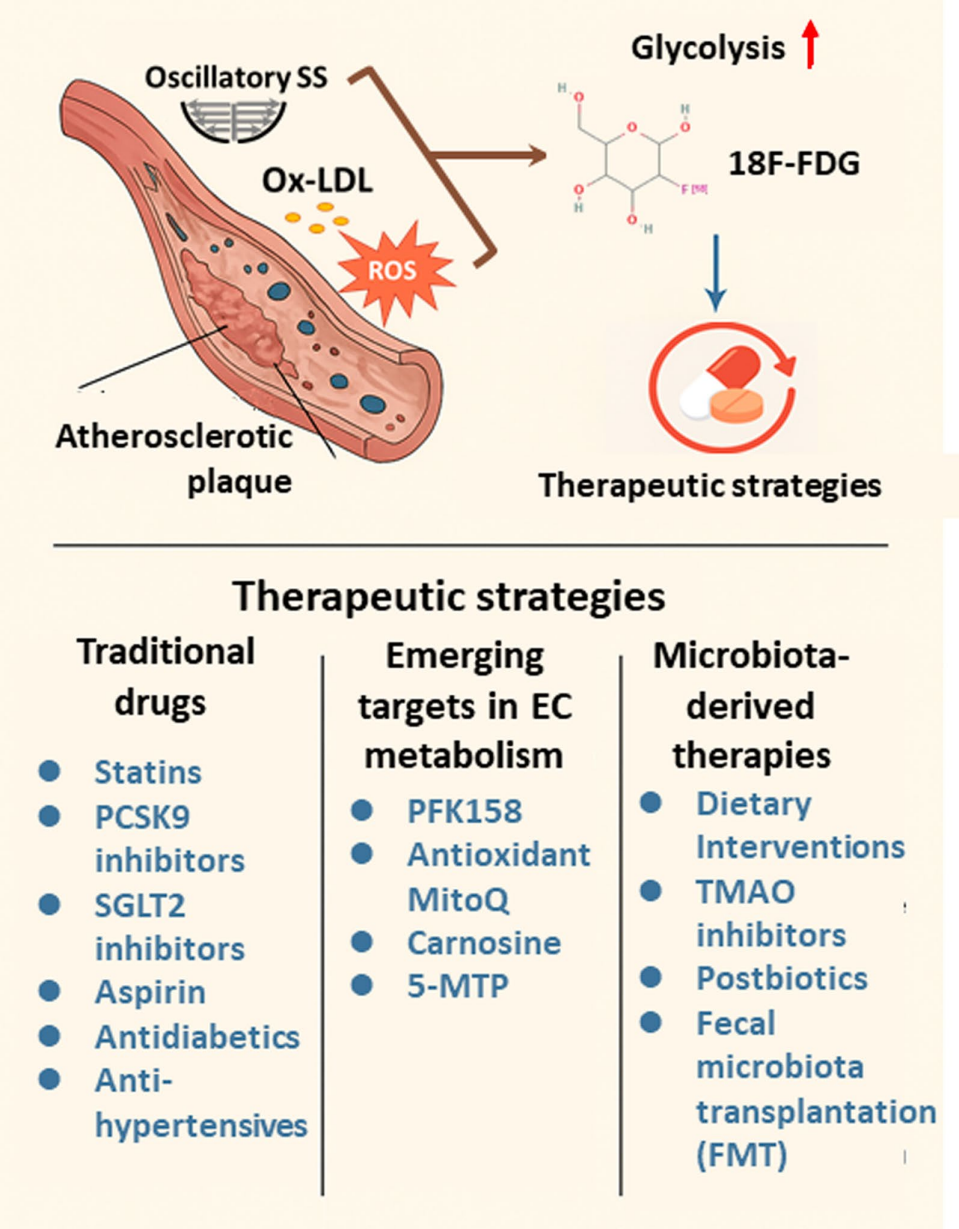
Targeting endothelial cell metabolism for atherosclerosis treatment. This figure illustrates how disturbed endothelial cell (EC) metabolism contributes to atherosclerosis development and outlines associated therapeutic strategies. Atherosclerotic plaque formation is initiated by factors, including aging-related influences, that promote glycolysis in ECs. The resulting increase in glucose uptake by ECs exhibiting high glycolytic activity can be visualized using ^18^F-FDG PET imaging to monitor treatment efficacy. The figure categorizes therapeutic strategies into three main areas: traditional drugs, emerging targets in EC metabolism, and microbiota-derived therapies

Figure 9 summarizes the key pathological mechanisms linking EC metabolic dysregulation to atherosclerosis and highlights three categories of therapeutic interventions—traditional drugs, emerging metabolic targets, and microbiota-derived therapies—currently under investigation.

## Conclusions

This review highlights EC metabolism as a fundamental determinant of vascular homeostasis in mechanosensitive and age-related atherosclerosis. Table 1 summarizes key metabolic enzymes and metabolites affected by aging or shear stress and which contribute to EC dysfunction and atherogenesis in multiple experimental models. ECs maintain a unique metabolic profile—characterized by a high rate of aerobic glycolysis—supporting a redox balance and NO production through the PPP/NADPH/eNOS axis. Aging and disturbed OS synergistically disrupt this metabolic equilibrium by impairing mitochondrial functions, increasing oxidative stress, and reprogramming cellular metabolism toward a pro-atherogenic state.

**Table 1:** Metabolic alterations of ECs in the onset and development of EC dysfunctions and atherosclerosis

EC metabolic pathway	Target enzyme or metabolite	Atherosclerotic risk factors	Affected signaling cascade	Experimental model (clinical or animal model)	EC dysfunction or atherosclerosis	Refs
Glycolysis	PDKs↓; PDC↑; LDHA↑	Aging		Cells: HUVECs	Senescence↑; mitochondrial ROS↑	[32]
	SLC2A1↑; HK2↑; PDK1↑	VEGF; disturbed flow (cone-and-plate viscometer); orbital flow (low: 5 dyn/cm^2^; high: 11 dyn/cm^2^)		Cells: HAECs; HUVECs; MAECs. Animal model: porcine aorta; ApoE^−/−^ mice with partial ligation of the left carotid artery; ApoE^−/−^ mice	EC proliferation↑; inflammation↑	[41, 221, 222]
	HK2↓	Atherosclerotic plaque vs. healthy arteries		Cells: HUVECs; HAECs; HMVECs; adipocytes; fibroblasts; cardiomyocytes; glial cells; hepatocytes; pericytes; neurons; dendritic cells. Human clinical sample: human carotid arteries	Atherosclerosis↑	[40]
	PFKFB3↑	Hypoxia; VEGF, lipoprotein(a); TGF-β; disturbed flow (cone-and-plate viscometer); orbital flow (low: 5 dyn/cm^2^; high: 11 dyn/cm^2^)	HIF-1α, VEGFA/VEGFR, pAKT	Cells: HUVECs; HAECs; EC cell lines (bEnd.3 and MS1); MAECs. Animal model: Ldlr^−/−^ mice treated with PFK158 and a high-cholesterol diet; PFKFB3^VEC−KO^ Ldlr^−/−^ mice with oxygen-induced retinopathy or tumor implantation; lipoprotein(a) Ldlr^−/−^transgenic mice; ApoE^−/−^ mice; porcine. Human clinical sample: human atheromatous carotid plaque; sera of patients with elevated lipoprotein(a)	EC proliferation↑, migration↑, inflammation↑, EndoMT↑, pathological angiogenesis↑, plaque area↑, vulnerable plaque↑	[22, 34, 35, 37, 39, 41, 221]
	Lactate↑	Aging; hyperglycemia; LPS; hypoxia; ox-LDL	p-AKT; ERK/calpain 1/2/VE-cadherin; lac-H3K18/SNAI1 promoter; lac-SNAI1	Cells: HUVECs; HCAECs; MAECs. Animal model: C57BL/6 J mice with CLP sepsis; EC-specific GFP-labeled C57BL/6 mice with myocardial infarction surgery; GPR81^−/−^ApoE^−/−^ mice. Human clinical sample: plasma and atherosclerotic arteries of patient with type 2 diabetes, cardiovascular disease, and carotid atherosclerosis	Permeability↑; EndoMT↑; pathogenic angiogenesis↑; atherosclerosis↑; cardiac fibrosis↑	[22, 32, 43–45, 53, 55, 56]
	Lactate↑	Exercise	Lac-Mecp2/Ereg/MAPK; GPR81/pERK5/KLF2	Cells: HUVECs; MAECs. Animal model: ApoE^−/−^ mice with exercise training	Mitochondrial ROS↓; inflammation↓; atherosclerosis↓	[57, 58]
	Acetate↑; ACSS2↑; acetyl-CoA↑	TGF-β	Acetyl-ALK5; acetyl-SMAD2/4	Cells: HUAECs. Animal model: Acss2^iECKO^, ApoE^−/−^ mice. Human clinical sample: aorta of patient with atherosclerosis	EndoMT↑; atherosclerosis↑	[60]
	G6PD (PPP) ↓	TGF-β	–	Cells: BAECs. Human clinical sample: plasma of patients with a known G6PD status and CVD	ROS↑; NO↓	[64, 68, 69]
	GFAT↑; glucosamine↑(HBP)	Hyperglycemia	IR/IRS/PI3K/Akt/eNOS	Cells: HCAECs; BVECs; BAECs; HMVECs; HUVECs; RCMVECs. Animal model: hyperglycemic ApoE^−/−^ mice. Human clinical sample: atherosclerotic arteries of type 2 diabetic patients	NO↓; ER stress↑; lipid accumulation↑; inflammation↑; vascular relaxation↓; atherosclerosis↑	[72–74]
	ALR↑, fructose↑, AGEs↑ (polyol pathway)	Hyperglycemia	MAPKP38/ERK1/2/eNOS	Cells: HCAECs. Animal model: ALR^+^ ApoE^−/−^ diabetic mice. Human clinical sample: plasma of diabetic patients with coronary artery atherosclerosis	NO↓; vasodilation↓; inflammation↑; atherosclerosis↑	[75, 77, 78]
Fatty acid metabolism	CD36↑	Aging; hyperlipidemia	–	Cells: HUVECs. Animal model: CD36^−/−^ LDL^−/−^ mice	Senescence↑; inflammation↑; atherosclerosis↑	[82, 91]
	FABP↑	Aging; hyperlipidemia; hyperglycemia	–	Cells: HUVECs; HMECs. Animal model: ApoE^−/−^ mice	Senescence↑; inflammation↑; lipid accumulation↑	[88, 90, 91]
	DGAT↑; ATGL↓; (LD formation)	Aging; hyperlipidemia	–	Cells: HUVECs; HAECs; LECs. Animal model: ApoE^−/−^ mice; ATGL^ECKO^ B6 mice with AAV-PCSK9 injection	Senescence↑; NO↓; vasodilation↓; blood pressure↑; cilia loss↑; inflammation↑; atherosclerosis↑	[91, 97–100]
	FASN↓	Aging; hyperglycemia	–	Cells: HUVECs; HVECs; LECs; COS cells. Animal model: C57BL/6 mice with diabetes and FASN inactivation	NO↓; permeability↑	[91, 108]
	Ceramides/S1P rheostat ↑	Aging	–	Cells: Wi38 fibroblasts; HUVECs	ROS↑; NO↓; permeability↑; atherosclerosis↑	[110, 111]
Amino acid metabolism	l-arginine/eNOS ↓; (GSH/GSSG↓; SAM/SAH↑; arginase↑; ADMA↑; BH_2_/BH_4_↑)	Aging; oxidative stress; hypertension	–	Cells: HAECs; BAECs. Animal model: WKY rats; SHR rats. Human clinical sample: serum of patients with atherosclerosis	NO↓; ROS↑; vasodilation↓	[118, 121, 130, 136, 138, 141]
	BCAAs↑	Aging	mTOR/pAMPK	Cells: HVECs; Human clinical sample: patients with coronary atherosclerosis	Mitochondrial ROS↑; inflammation↑; atherosclerosis↑	[151, 155]
Mitochondrial respiration	Mitochondrial mass, component and respiration↓	Aging; disturbed flow (cone-and-plate viscometer)	–	Cells: HAECs. Animal model: Fisher 344 rats; porcine aorta	Mitochondrial ROS ↑; inflammation↑	[162, 163, 222]
Circulating metabolites	AA metabolism	Aging	–	–	Vasodilation↓; inflammation↑	[176]
	β-alanine/carnosine ↓	Aging; disturbed flow (parallel-plate flow, PS, 12 ± 4 dynes/cm^2^ or OS, 0.5 ± 4 dynes/cm^2^)	Nrf2/Ho-1	Cells: HaCaT; HOECs; HUVECs. Animal model: C57BL/6 mice; ApoE^−/−^ mice; Rosa26-mTmG mice with partial carotid ligation; Dnmt1^ECKO^ C57BL/6 mice with partial ligation and AAC-PCSK9 injection. Human clinical sample: blood of young and older humans	Senescence↑; ROS↑; DNA damage↑; inflammation↑; EndoMT↑; atherosclerosis↑	[179, 180, 183, 232]
	5-MTP ↓	LPS; TNF-α	p38 MAPK	Animal model: C57BL/6 mice with LPS injection, CLP, or femoral artery denudation injury. Human clinical sample: patients with sepsis syndrome	Systemic inflammation↑; restenosis caused by arterial injury↑	[184–186]
Gut microbiota-derived metabolites	TMAO ↑	Diet; gut microbiota; aging	–	Animal model: C57BL/6 N mice. Human clinical sample: blood of young and older humans	NO↓; ROS↑; vasodilation↓; atherosclerosis ↑	[187, 189]

This table summarizes key findings on how alterations in metabolic pathways in ECs contribute to EC dysfunction and the progression of atherosclerosis, particularly in the context of aging, shear stress, and other various atherosclerotic risk factors. *PDKs* pyruvate dehydrogenase kinases, *PDC* pyruvate dehydrogenase complex, *LDHA* lactate dehydrogenase A, *SLC2A1* glucose transporter type 1, *HK2* hexokinase 2, *PFKFB3* 6-phosphofructo-2-kinase/fructose-2,6-bisphosphatase 3, *ACSS2* acyl-CoA synthase short chain 2, *G6PD* glucose-6-phosphate dehydrogenase, *PPP* pentose phosphate pathway, *GFAT* glutamine:fructose-6-phosphate amidotransferase, *HBP* hexosamine biosynthesis pathway *ALR* aldose reductase, *AGEs* advanced glycation end products, *FABP* fatty acid-binding protein, *LD* lipid droplet, *DGAT1* diacylglycerol O-acyltransferase 1, *ATGL* adipose triglyceride lipase, *FASN* fatty acid synthase, *S1P* sphingosine-1-phosphate, *GSH* glutathione, *GSSG* glutathione disulfide, *SAM* S-adenosylmethionine, *SAH* S-asenosylhomocysteine, *ADMA* asymmetric dimethylarginine, *BH*_*2*_ dihydrobiopterin, *BH*_*4*_ tetrahydrobiopterin, *BCAAs* branched-chain amino acids, *AA* arachidonic acid, *5-MTP* 5-methoxytryptophan, *TMAO* trimethylamine N-oxide, *HIF-1α* hypoxia-inducible factor-1α, *pAKT* phosphorylated protein kinase B, *ERK* extracellular signal-regulated kinase, *lac-H3K18* histone H3 lysine 18 lactylation, *CLP* cecal ligation and puncture, *GPR81* G protein-coupled receptor 81, *MeCP2* methylated CpG-binding protein 2, *Ereg* epiregulin, *MAPK* mitogen-activated protein kinase, *ALK5* activin receptor-like kinase 5, *HUVECs* human umbilical vein endothelial cells, *BVECs* bovine venular endothelial cells, *BAECs* bovine aortic endothelial cells, *HMVECs* human microvascular endothelial cells, *RCMVECs* rat coronary microvascular endothelial cells, *LECs* lung endothelial cells, *HaCaTs* human dermal keratinocytes, *HOECs* human oral epithelial cells

Aging and shear stress jointly orchestrate profound and context-dependent reprogramming of EC metabolism. Core transcriptional regulators, such as KLF2/4 and HIF-1α, integrate mechanical and hypoxic cues to regulate metabolic adaptations. LS activates KLFs to promote metabolic quiescence and antioxidant defense, whereas OS and aging might reduce KLF expression while stabilizing HIF-1α, thereby driving glycolytic activation, mitochondrial suppression, and inflammation. In parallel, AMPK and YAP/TAZ act as mechanosensitive metabolic regulators–with AMPK maintaining energy balance and mitochondrial homeostasis, and YAP/TAZ enhancing glycolysis and EC proliferation under disturbed flow. Aging impairs these stress-responsive axes, which weakens EC adaptation to mechanical stress and promotes dysfunction.

Beyond glycolysis and mitochondrial respiration, dysregulated FA oxidation, aa metabolism (e.g., l-arginine, glutamine, and BCAAs), and a NAD⁺ redox imbalance contribute to senescence-associated endothelial phenotypes in the aging vasculature. Moreover, circulating and microbiota-derived metabolites, particularly the pro-atherogenic TMAO, further amplify oxidative stress and inflammation, linking systemic metabolic alterations with local endothelial pathologies. These overlapping influences converge on shared molecular nodes such as PFKFB3, eNOS, ADMA, ROS, and BH_4_/BH_2_, whose dysregulation reflects and reinforces vascular aging and atherosclerosis.

**Fig. 10:**
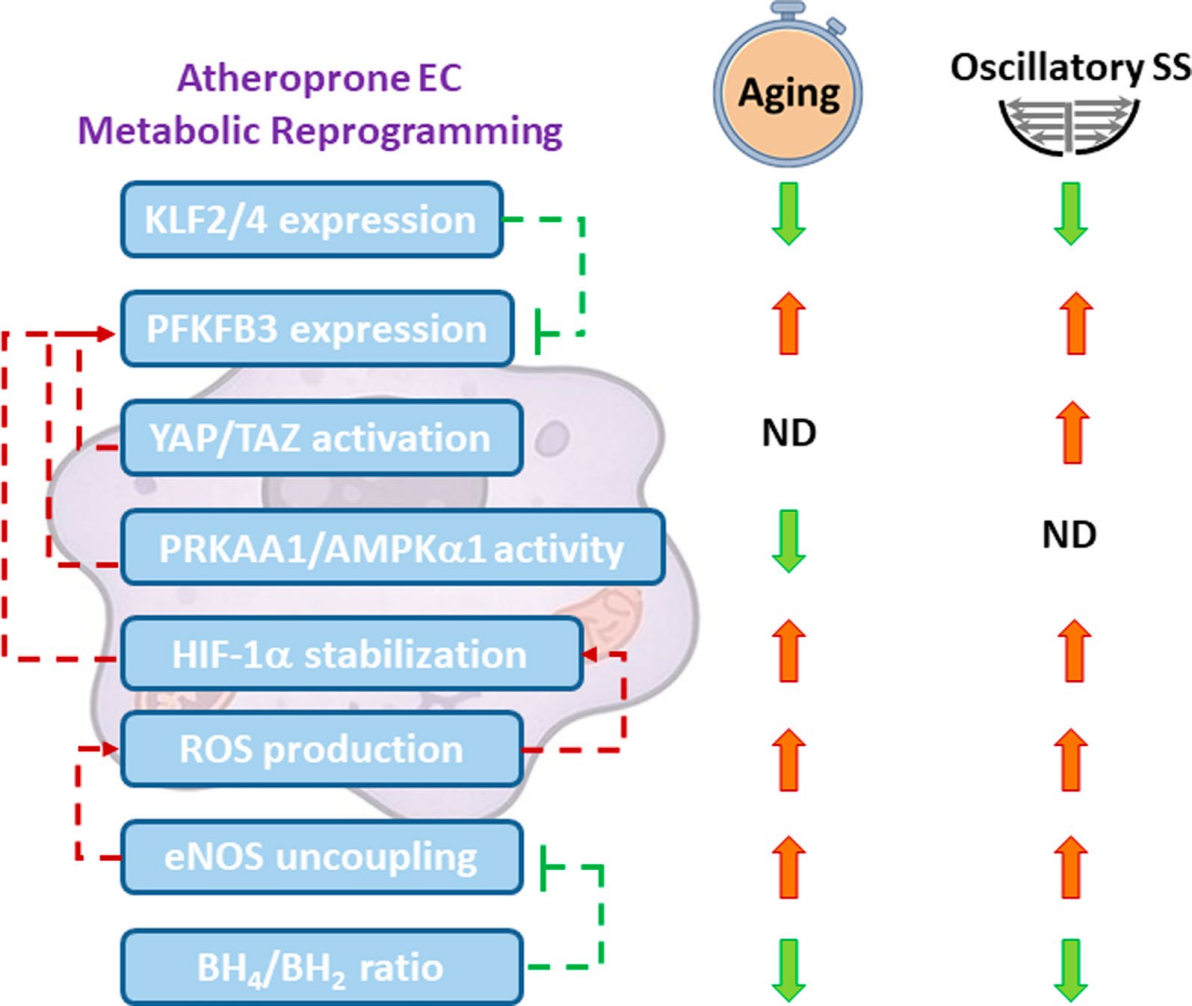
Aging and oscillatory shear stress induce atheroprone EC metabolic reprogramming. This schematic illustrates how both aging and oscillatory shear stress (OSS) drive critical molecular changes associated with metabolic reprogramming in ECs, ultimately contributing to atheroprone phenotypes. These two factors commonly lead to a decrease in KLF2/4 expression and an increase in PFKFB3 expression, a key enzyme in glycolysis. Furthermore, aging and OSS disrupt the delicate balance of BH4/BH2, essential cofactors for eNOS activation, resulting in eNOS uncoupling and subsequent increased reactive oxygen species (ROS) production and enhanced HIF-1α activity. While it is known that HIF-1α is activated by both aging and OSS, the precise impacts on PRKAA1/AMPKα and YAP/TAZ signaling remain an area of ongoing research and debate. Nevertheless, the activated PRKAA1/AMPKα, YAP/TAZ, and HIF-1α pathways all converge to promote PFKFB3 expression, further solidifying the shift towards glycolytic metabolism. The colored arrows within the diagram visually represent the direction of these changes (↑ indicating an increase and ↓ indicating a decrease), while dashed lines highlight the intricate interconnections among these various pathways. ND, not determined

Figure 10 summarizes the major metabolic regulators collaboratively affected by aging and fluid shear stress, illustrating how their integration might drive endothelial dysfunction. The advent of single-cell transcriptomics, proteomics, and metabolomics technologies now enables unprecedented resolution in analyzing EC heterogeneity and metabolic dynamics in vivo. Future studies combining these approaches with disease models of aging and disturbed flow should enable precision mapping of vascular metabolic remodeling and uncover novel targets to delay or prevent atherosclerosis in aging populations.

## Data Availability

No datasets were generated or analysed during the current study.

## Abbreviations

AA: Arachidonic acid
ACE: Advanced glycation end product
AMPK: AMP-activated protein kinase
ARD: Age-related disease
BCAA: Branched-chain amino acid
CAD: Coronary artery disease
CVD: Cardiovascular disease
EC: Endothelial cell
EndoMT: Endothelial-to-mesenchymal transition
Enos: Endothelial nitric oxide synthase
ER: Endoplasmic reticulum/reticular
FA: Fatty acid
FAO: Fatty acid oxidation
FOXO1: Forkhead box O1
GLUT: Glucose transporter
GPR81: G-protein-coupled receptor 81
HBP: Hexosamine biosynthesis pathway
HCR: Hydroxy methylglutaryl coenzyme A reductase
HIF: -1α, Hypoxia-inducible factor 1-alpha
HK: Hexokinase
KLF: Krüppel-like factor
LD: Lipid droplet
LDH: Lactate dehydrogenase
LS: Laminar shear stress
MAPK: Mitogen-activated protein kinase
5-MPT: 5-Methoxytryptophan
NO: Nitric oxide
OS: Oscillatory shear stress
OXPHOS: Oxidative phosphorylation
PDK1: Pyruvate dehydrogenase kinase 1
PDH: Pyruvate dehydrogenase
PFK: Phosphofructokinase
PFKFB: 3, 6-Phosphofructo-2-kinase/fructose-2,6-bisphosphatase 3
PPP: Pentose phosphate pathway
ROS: Reactive oxygen species
SASP: Senescence-associated secretory phenotype
S1P: Sphingosine-1-phosphate
SCFA: Short-chain fatty acid
TMAO: Trimethylamine-N-oxide
